# Sidewall Patterning in 3D Micro/Nanosystems: A Review

**DOI:** 10.3390/mi17090992

**Published:** 2026-08-22

**Authors:** Xinchuan Liu, Cheng Luo

**Affiliations:** 1 Department of Engineering and Industrial Professions, University of North Alabama, Florence, AL 35632, USA; 2 Department of Mechanical and Aerospace Engineering, University of Texas at Arlington, Arlington, TX 76019, USA

**Keywords:** sidewall patterning, hot embossing, shape memory polymers, flexible shadow masks, 3D micro/nanosystems

## Abstract

Current micro/nanosystems mainly rely on a planar fabrication framework, where structures are built layer-by-layer on flat surfaces. This conventional approach leaves vertical sidewalls underutilized, posing geometric limits in packaging density, three-dimensional (3D) interconnects, and multi-surface functionalization. To overcome these constraints, sidewall patterning has emerged as a promising strategy, enabling 3D integrated circuits, templates for directed nanostructure synthesis, and microfluidic drag reduction. Nevertheless, traditional photolithography and non-photolithographic techniques face challenges when applied to vertical or curved 3D surfaces. Unidirectional radiation and restricted focal depths prevent high-fidelity pattern transfer, even when using soft lithography, scanning probes, or nanoimprinting. To address these geometric and mechanical barriers, our group has developed several approaches for patterning the sidewalls of microsystems, which are the primary focus of this review. Building upon our approaches, this review further surveys related sidewall-patterning strategies, including micro-transfer printing, multi-stimuli-responsive mechanics, block copolymer self-assembly, two-photon polymerization, and laser-induced forward transfer. Collectively, these techniques expand the capabilities of sidewall engineering and provide valuable insights into next-generation 3D micro- and nanomanufacturing.

## 1. Introduction and Motivation

### 1.1. Historical Limitations of the 2D Planar Paradigm

Over the past few decades, the rapid expansion of microelectronics, microelectromechanical systems (MEMSs), and microfluidics has been fundamentally anchored in the successful evolution of the conventional planar fabrication framework. Within this traditional 2D manufacturing architecture, the construction of fine features, pattern transfers, and functional device integrations is highly confined to the flat top surface planes of substrates. To fabricate complex systems on a common substrate, conventional methods rely on stacking multiple layers of planar features sequentially. This fabrication process generally involves multiple cycles of lithography, anisotropic etching, and thin-film deposition [1,2].

**Figure 1:**
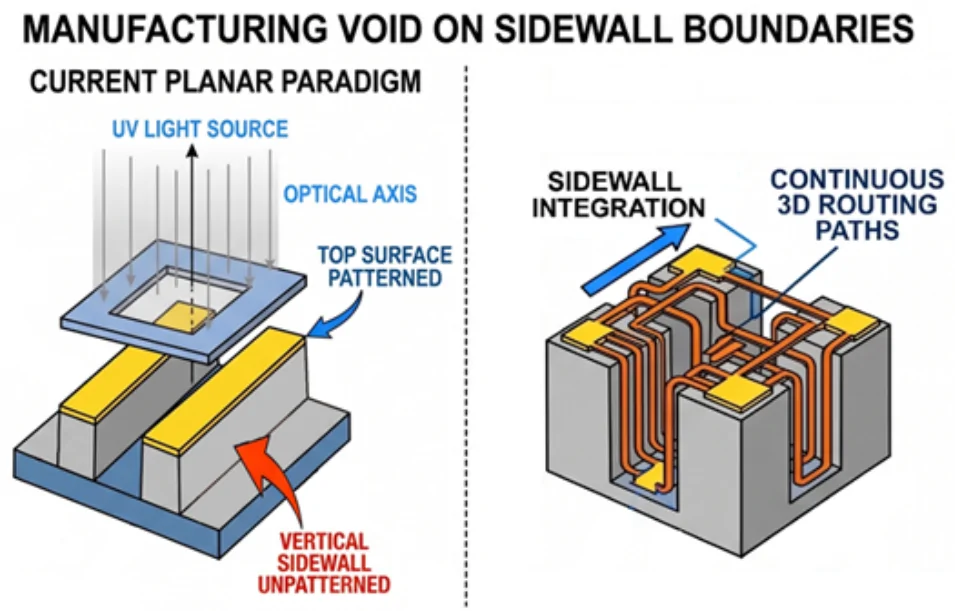
Conceptual transition from conventional planar architecture to 3D multi-surface sidewall integration. (**Left**) Inherent limitations of the conventional 2D framework, where collimated light fields or linear deposition fluxes restrict pattern transfer to horizontal planes, leaving vertical sidewalls unpatterned (blank space). (**Right**) Sidewall integration, where continuous circuit traces cross rigid step boundaries and embed high-density functional components (e.g., *in situ* serpentine trench resistors) along vertical walls, markedly enhancing packaging density for 3D integrated circuits.

However, as the demanding metrics of device miniaturization, functional diversification, and system-in-package spatial efficiency continuously escalate, this classical method encounters limitations [3,4]. This is primarily because it was established on flat horizons. Due to the lack of effective technological pathways to manipulate and transfer high-fidelity patterns onto vertical interfaces, the sidewall boundaries have long remained a technological challenge (Figure 1). This historical blind spot substantially restricts the geometric density of material and energy transport lines in 3D space, leaving immense surface areas largely unutilized for functional device integration [5].

### 1.2. Definition of Sidewall Patterning

In this review, “sidewall patterning” refers to the fabrication of micro- and nanopatterns on the pre-existing non-planar surfaces of micro/nanosystems. These surfaces include:

(i) Vertical and sloped sidewalls within deep trenches, microchannels, and high-aspect-ratio silicon/polymer cavities;
(ii) Curved closed surfaces, including cylindrical outer walls and conical tips;
(iii) Non-developable geometries, such as micropipettes with curved, conical tips.

The first two types of surfaces are *developable*. A developable surface is a surface that can be unrolled or flattened onto a plane without stretching, compressing, or tearing it. In differential geometry, an ideal developable surface has zero Gaussian curvature everywhere (apart from possible singular points). In contrast, non-developable surfaces possess nonzero Gaussian curvature and cannot be flattened without distortion.

#### 1.2.1. 3D Integrated Circuits and New Vertical Interconnect Architectures

Traditional interlayer interconnects, such as through-silicon vias in semiconductor packaging, require multi-step, time-consuming processing. These methods depend heavily on deep reactive-ion etching (DRIE), dielectric/seed-layer deposition, and electroplating [6,7]. This structure requires high capital investment and complex yield management, while presenting inherent risks related to stress concentration and thermal expansion mismatch [8].

By introducing sidewall patterning techniques, continuous conductive wiring tracks or localized microdevices can be directly created on the vertical surfaces of pre-existing micro-protrusions, such as rigid Si posts, polymeric micro-pedestals, and trench walls. As schematized in the manufacturing layout (Figure 1), these flexible interconnect circuits can initiate from one side of a micro-topography and traverse vertically along the sidewall face. They then cross the top horizontal plane and extend to the opposite side. This operational mode directly utilizes existing structural sidewalls as 3D electrical pathways. Consequently, this approach reduces manufacturing reliance on through-chip interconnect holes. By streamlining the signal path, it would minimize propagation delays and offer an effective design for high-density integrated circuit packaging. In addition, functional components, such as microresistors [9], could be fabricated directly on sidewalls to improve the performance of the corresponding microsystems.

#### 1.2.2. Functional Templates for Directed Horizontal Synthesis of 1D Nanostructures

**Figure 2:**
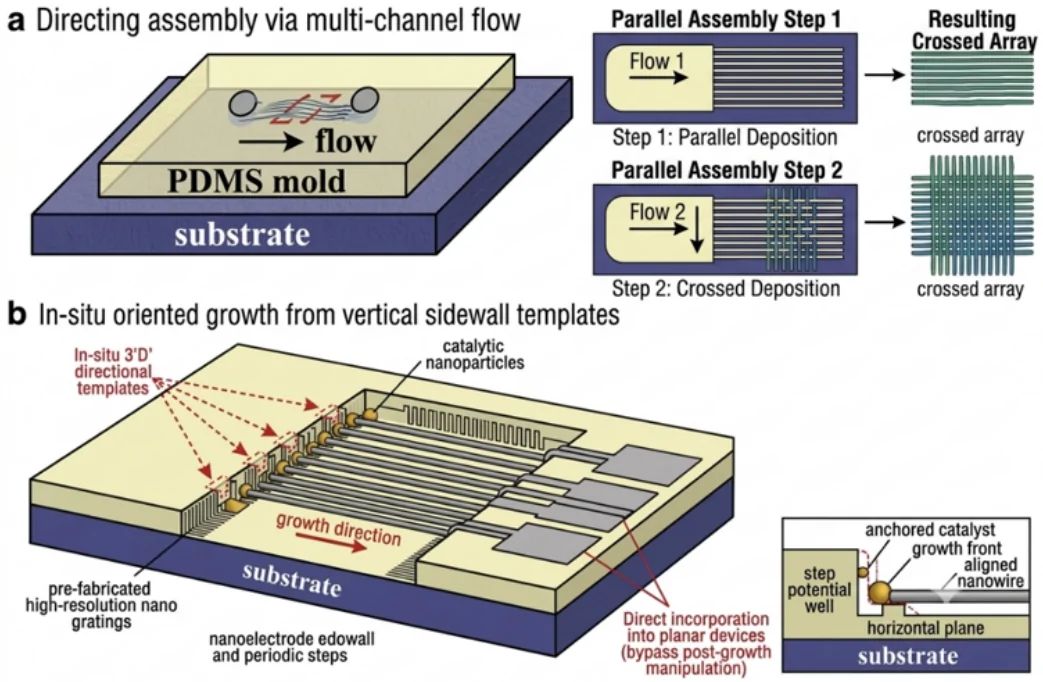
Comparison of directed assembly and *in situ* synthesis strategies for 1D nanostructures. (**a**) Fluidic assembly scheme directing parallel and crossed nanomaterial arrays using alternating fluidic shear flows in a multi-channel PDMS mold (adapted from [11]). (**b**) *In situ* oriented growth from vertical sidewall templates. Prefabricated micro/nano-gratings or periodic steps establish physical/chemical potential wells that precisely anchor catalytic nanoparticles, inducing and confining horizontal directed growth along the sidewall planes (adapted from [12]). This inherent alignment enables direct incorporation into planar device architectures without post-growth manipulation. Insert: Cross-sectional view of the step potential well anchoring the catalyst at the growth front. (**a**) Adapted from [11]; (**b**) Adapted from [12], licensed under CC BY 4.0. Permission obtained.

1D nanomaterials like nanowires and nanotubes offer high electron mobility, mechanical flexibility, and large specific surface areas. These properties make them key components in advanced field-effect transistors and sensitive biosensors [10]. However, within existing wafer-scale catalytic growth, these 1D features are normally synthesized longitudinally along vertical directions through top or bottom planar templates. To integrate these 3D nanowires or nanotubes into the functional circuit networks of planar platforms, post-processing efforts are often deployed to reorient them horizontally (Figure 2a; [11]). These methods include alternating electric fields, precise magnetic alignments, or fluidic shear flows, which frequently face yield limitations and risk mechanical damage.

If high-resolution micro/nano-gratings, periodic steps, or porous tracks are fabricated directly on the vertical sidewalls of a planar system, these patterns can serve as built-in, *in situ* 3D directional templates (Figure 2b). The spatial potential wells established by these sidewall features precisely anchor catalytic nanoparticle sites, inducing and confining the horizontal directed growth of nanowires or nanotubes along the sidewall planes [12]. Because their synthesis direction is inherently parallel and aligned with the host planar plane from the onset, they can be directly incorporated into planar device architectures. This bypasses complex post-growth mechanical manipulation and alignment assemblies, securing a substantial simplification in the scalable manufacturing of nanoelectronics.

#### 1.2.3. Multi-Surface Multi-Dimensional Wetting Control and Fluidic Drag Reduction

**Figure 3:**
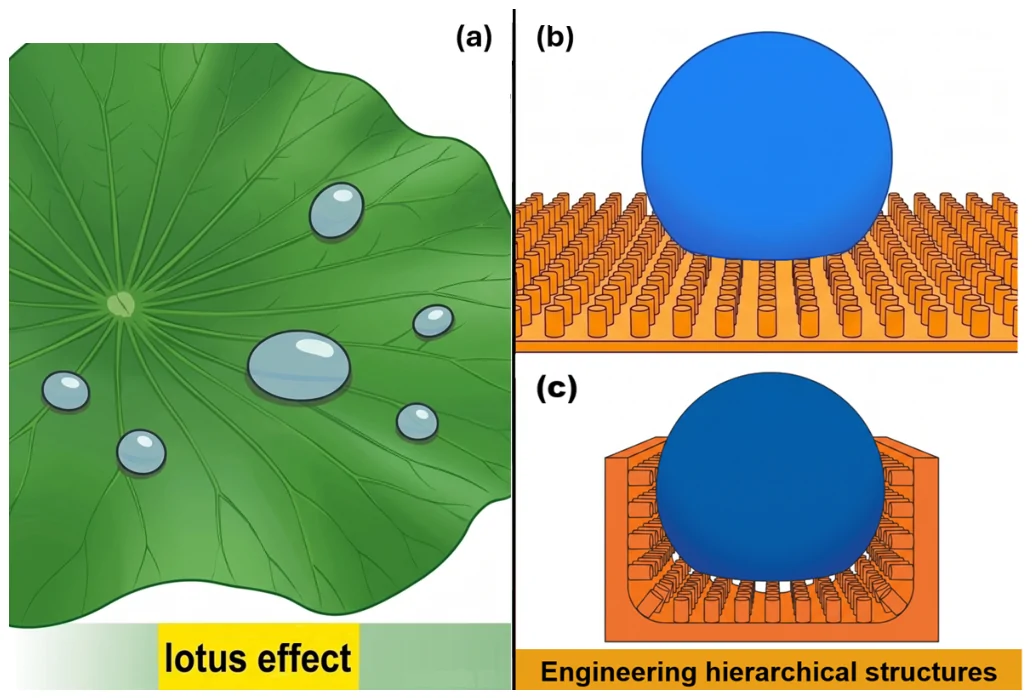
Bio-inspired superhydrophobic surfaces and engineering of the Cassie–Baxter wetting state. (**a**) The natural “lotus effect” observed on a lotus leaf surface, demonstrating dynamic water mobility and self-cleaning behavior driven by hierarchical surface micro/nanotopography. (**b**) Schematic illustration of a hierarchical artificial surface in a stable Cassie–Baxter state. High-aspect-ratio micropillar arrays robustly trap a continuous air cushion beneath the liquid. This structure supports a spherical water droplet and mimics natural superhydrophobic mechanisms, thereby minimizing viscous shear resistance across functional device surfaces. (**c**) Incorporation of micropillars on the channel sidewalls to reduce sidewall drag.

Natural superhydrophobic and self-cleaning surfaces, such as the lotus leaf (Figure 3a), have inspired new approaches in fluid mechanics. Applying micro/nanotopographical engineering to modulate interfacial hydrodynamics has since become a key area of microfluidics [13]. As a working fluid flows across a hydrophobic, periodic micro-topography, such as a micropillar array, within a Cassie–Baxter state, the gaps between the features stably trap a continuous gas cushion. This plastron physically isolates the moving liquid from the solid substrate, introducing a low shear-stress slip boundary condition at the solid-liquid interface. This effect substantially reduces viscous shear resistance and lowers the input pumping power required to actuate the fluid (Figure 3b; [14]).

Nevertheless, conventional drag-reduction surface engineering within microfluidic channels faces a spatial limitation: topographical modifications are traditionally confined to the flat bottom plane of the channel. In laminar microfluidic streams, the moving liquid experiences viscous shear friction not only from the horizontal floor but equally from the two vertical sidewalls. Exploiting sidewall patterning technologies allows researchers to address this limitation, enabling a holistic, multi-surface fabrication protocol. This approach integrates flexible polymeric micropillar arrays with accurately tuned aspect ratios onto both the floor and two vertical sidewall surfaces of deep channels (Figure 3c; [15]). This continuous, multi-surface superhydrophobic coating cooperatively alters the local interfacial boundary conditions across all contact planes, compressing global fluidic flow resistance and driving pressure requirements.

### 1.3. Literature Search and Selection Methodology

To evaluate sidewall patterning technologies comprehensively, a literature search was conducted across core scientific databases, including Web of Science, IEEE Xplore, Scopus, and PubMed. The survey primarily focused on peer-reviewed articles and reviews published between 2000 and 2026.

The primary search strategy utilized targeted keyword combinations, including: “sidewall patterning”, “non-planar fabrication”, “3D/curved surface lithography”, “transfer printing”, “shape memory polymers”, “flexible shadow masks”, “direct-write patterning”, “3D microfluidic routing”, and “conformal electronics”. Following the initial topic identification, publications were qualitatively screened for technological relevance, physical mechanisms, and applicability to sidewall patterning. To provide necessary historical context and foundational principles, several classic pre-2000 studies (e.g., foundational works on soft lithography and early non-photolithographic concepts) were manually identified and included via backward reference tracking. This approach aims to provide a mechanics-driven, balanced synthesis of physical mechanisms and technological evolution in sidewall engineering.

### 1.4. Classification Criteria and Outline of the Review

Fabrication routes in micro/nanosystems can be broadly classified from two perspectives. From the standpoint of material processing, they are generally divided into subtractive and additive approaches, depending on whether material is removed from or deposited onto the substrate. Alternatively, they can be classified as direct physical/chemical modification and pattern relocation [5], according to whether patterns are formed directly on the target substrate or fabricated elsewhere and then transferred to the target surface. Since the central objective of sidewall patterning is to create patterns on non-planar sidewall surfaces, the latter classification more clearly distinguishes the underlying patterning mechanisms and is therefore adopted in this review. As our group has developed multiple approaches for sidewall patterning in both categories of pattern relocation and direct physical/chemical modification [9,15,16,17,18,19,20], this review will start and focus on our approaches, followed by related work done by other researchers.

Conformal technologies such as chemical vapor deposition, physical vapor deposition, atomic layer deposition, and Bosch-process fluorocarbon passivation provide valuable means of surface modification. Because these processes generally produce continuous surface coverage without defining localized, discrete geometries, they are not considered standalone sidewall-patterning techniques in this review. Instead, they are treated as auxiliary surface or boundary treatments. Spatially selective chemical or wettability modification, such as localized plasma treatment, can alter surface properties and may subsequently facilitate the formation of sidewall patterns. Such surface modifications alone are classified as sidewall-patterning approaches in this review.

The outline of this review is as follows. Section 2 dissects the fundamental depth-of-focus limitations and geometric failures of conventional planar lithographic architectures when confronted with vertical sidewall topologies. Section 3 focuses on the approaches that use flexible intermediate membranes for pattern relocation. Section 4 details the thermo-responsive strain-recovery mechanics of shape-memory polymers and multi-stimuli-responsive techniques. Section 5 decodes the flexible membrane-mediated stenciling within deep trenches, as well as block copolymer self-assembly. Section 6 centers on compliance-driven conformal membrane transfers and gas-flux-assisted non-destructive printing onto non-developable, fragile high-curvature surfaces. Section 7 reviews photon-energy-driven digital maskless approaches, including sub-diffraction two-photon polymerization and laser-induced forward transfer. Finally, Section 8 constructs a manufacturing comparison matrix, isolates key remaining techno-economic bottlenecks, offers prospective insights into next-generation hybrid fabrication systems, and concludes this review.

The methods reviewed in Section 3 and Section 4 are classified as pattern relocation techniques because they transfer pre-fabricated patterns from a donor substrate to the target sidewalls. In contrast, the approaches described in Section 5, Section 6 and Section 7 are categorized as direct physical/chemical modification, as the patterns are created directly on the sidewall surfaces. The use of auxiliary elements, such as shadow masks, does not alter this classification because they serve only to assist pattern definition rather than to relocate pre-existing patterns.

## 2. Limitations of Conventional Lithographic Architectures

### 2.1. Directional Exposure and Depth-of-Focus Constraints of Photolithography

Traditional micro/nanofabrication industries rely heavily on light- or particle-beam-based lithography, such as ultraviolet, electron-beam, and X-ray lithography. Conventionally, the fundamental optical configuration of these planar lithographic processes relies on vertical beam exposure. In this alignment, the energy propagation path remains orthogonal to the nominal substrate plane, primarily confining photochemical cross-linking or surface modification to top horizontal boundaries [21,22].

When confronted with high-aspect-ratio 3D structures, such as vertical channels, micropillar arrays, or deep trenches, this directional exposure mechanism encounters a critical geometrical limitation [23]. Vertical sidewalls align parallel to the propagation direction of the incident beam. Consequently, vertically impinging photons or particles cannot be effectively intercepted or absorbed by the photoresist coating on these boundaries. Consequently, the photoresist along the sidewall fails to accumulate the threshold exposure dosage, making pattern transfer physically challenging.

Optical exposure systems are stringently bounded by the physical limits of the depth of focus (*DOF*). The *DOF* defines the vertical spatial window over which the lithographic projection can maintain sharp imaging, mathematically governed by Rayleigh’s equation [22,24]:

(1) $$\mathrm{DOF}=k_{2}\frac{\lambda }{{\mathrm{NA}}^{2}}$$

where λ is the incident wavelength, *NA* represents the numerical aperture of the optical system, and $k_{2}$ is a process-dependent factor. In micro/nanofabrication pursuing high resolution at the micro- or nanoscale, maximizing the *NA* is universally required, which inherently forces the *DOF* to shrink drastically to a few micrometers or even sub-micrometer regimes. However, the sidewall heights or trench depths of typical 3D micro-topographies frequently scale up to tens or hundreds of micrometers, far exceeding the operational depth window of the lithographic system.

Focusing on the top planar surface induces substantial blurring and structural distortion near the substrate base and deep sidewalls, driven by diffraction and defocusing [25]. This focus depth bottleneck prevents traditional photolithography from traversing discontinuous 3D topology at high resolution. Consequently, it impairs pattern continuity, inherently causing circuit line breaks or fabrication failures at multi-planar corner junctions.

### 2.2. Geometric and Mechanical Failures of Planar Non-Photolithographic Approaches

To bypass the reliance of traditional photolithography on optical collimation equipment and demanding chemical resists, a series of classical non-photolithographic approaches have been developed. These methodologies primarily include dip-pen nanolithography [26], soft lithography [27], and nanoimprint lithography [28]. Although they exhibit low-cost, large-area manufacturing capabilities on flat 2D horizons, they encounter geometric and mechanical bottlenecks when applied to vertical sidewall patterning:

#### 2.2.1. Spatial Constraints of Scanning Probe Lithography (Dip-Pen)

Direct-writing approaches typified by dip-pen nanolithography rely heavily on the micro-cantilever probe tip of an atomic force microscope acting as a transfer vector to deliver molecular inks *in situ* onto a substrate surface [26]. However, the mechanical degrees of freedom of this high-precision structure are confined to the horizontal *X–Y* plane. In addition, the probe tip features symmetric conical or pyramidal geometry [29].

When the writing assembly encounters a vertical sidewall or an inclined 3D feature, the underlying cantilever body is prone to physical collisions with the flat top plane or protruding edges of the microstructure. This spatial interference physically blocks the probe tip from approaching or making contact with the vertical boundary [30]. Cantilever sensors are calibrated to detect and feed back minute force deflections exclusively along the vertical *Z*-axis. Consequently, sideways contact with a vertical wall leaves the system unable to regulate the precise lateral forces required for scanning. Such localized contact typically leads to mechanical fracturing of the fragile tip or tracking ink contamination, tightly trapping its fabrication domain to horizontal top platforms [29,31].

#### 2.2.2. Contact Mechanics Failures of Soft Lithography Across Discontinuous Topology

Soft lithography, typified by microcontact printing, relies on compliant elastomeric stamps, most commonly poly(dimethylsiloxane) (PDMS). Its core mechanism requires establishing molecular-level conformal contact with a substrate to execute chemical ink transfer. This protocol requires applying a uniform normal contact stress directed perpendicular to the matching interfaces [27].

Rigid substrates often feature discontinuous topologies, such as high-aspect-ratio vertical channels, trenches, or micropillars. During physical downward embossing, these features subject the flexible elastomeric stamp to substantial geometric trapping. Stamp deformation is primarily driven by macroscale normal loads along the vertical direction. Consequently, it cannot generate the necessary lateral compressive stress to conformally match vertical sidewalls. This mechanical deficit creates microscopic physical gaps between the stamp and the sidewalls, preventing the formation of molecular conformal contact. Driven by localized stress concentrations, the soft stamp is prone to local elastic buckling or roof collapse when crossing sharp structural boundaries, such as the top vertex of a trench wall [23,32]. This mechanical mismatch not only fails to transfer patterns onto the sidewall face but instead generates substantial pattern distortion and ink blurring across adjacent horizontal horizons.

#### 2.2.3. Normal Loading Transmission Deficit of Nanoimprint Lithography

Nanoimprint lithography, recognized as a high-resolution, high-throughput non-photolithographic process, forces fine features mechanically into a thermally softened or photo-curable polymeric thin film using rigid or semi-rigid hard molds [28]. The classical hydrodynamic model of conventional nanoimprint lithography assumes fluid squeezing between parallel, planar boundaries. Under these unidirectional normal loading conditions, pattern replication relies on generating local thickness contrast.

Conventional imprinting molds feature flat bottom topologies facing the substrate, directing substantial mechanical compressive loads along the vertical direction. According to the fundamental principles of solid mechanics, when evaluating force transmission along a non-planar boundary, the net normal stress component $\sigma_{n}$ acting on an interface is restricted by the resolved component equation:

(2) $$\sigma_{n}=\sigma_{Z}\cos\left(\theta \right)=\sigma_{Z}\cos\left(90{}^{\circ}\right)=0,$$

where $\sigma_{n}$ is the net interface normal stress, $\sigma_{z}$ is the applied vertical compressive stress, and θ represents the angle between the vertical loading vector and the normal vector of the interface. Essentially, when confronted with a vertical sidewall, the interface normal vector runs perpendicular to the vertical loading profile (θ=90°). Because cos(90°)=0, the unidirectional vertical imprinting load yields a zero-net normal stress component across the vertical surface boundaries ($\sigma_{n}=0$).

Consequently, the vertical imprinting load is incapable of translating into the lateral stress fields mandatory to force the softened or curing polymers to flow horizontally and fill the micro-grooves along vertical sidewalls [33,34]. Deprived of this lateral driving force, the material under softened states cannot achieve high-fidelity replication of lateral micro/nano-features from the mold onto the vertical walls. This unidirectional loading transmission deficit inherently deprives conventional nanoimprint lithography of the mechanical capacity to pattern high-aspect-ratio vertical surfaces.

A summary of the physical mechanisms and specific failure modes associated with these classical patterning technologies when applied to non-planar 3D topographies is provided in Table 1.

## 3. Pattern Relocation via Flexible Membranes

One of the primary strategies for sidewall patterning is to relocate pre-fabricated patterns from planar surfaces to sidewall surfaces. This section reviews relocation methods based on hot embossing and the mechanical folding of flexible membranes. The next section discusses an alternative relocation approach that exploits the strain recovery of shape-memory polymers to transfer patterns onto sidewall surfaces.

### 3.1. Hot-Embossing-Based Pattern Relocation

In macroscale mechanical manufacturing, standard punching via hot embossing has over a century of industrial use for sheet metal fabrication. This method shapes localized structural topographies primarily through mechanical tool–die interactions. To adapt these macroscopic punching principles to the micro/nanofabrication domain, micropunching lithography was developed as an embossing-based patterning route [35,36,37,38,39,40]. This methodology enables deterministic control and scalable production of complex 3D microstructures using diverse functional materials [15,41,42]. Inheriting its macroscopic lineage, the MPL process infrastructure dynamically comprises two core operational modalities: the “cutting” operation and the “drawing” operation [40].

**Figure 4:**
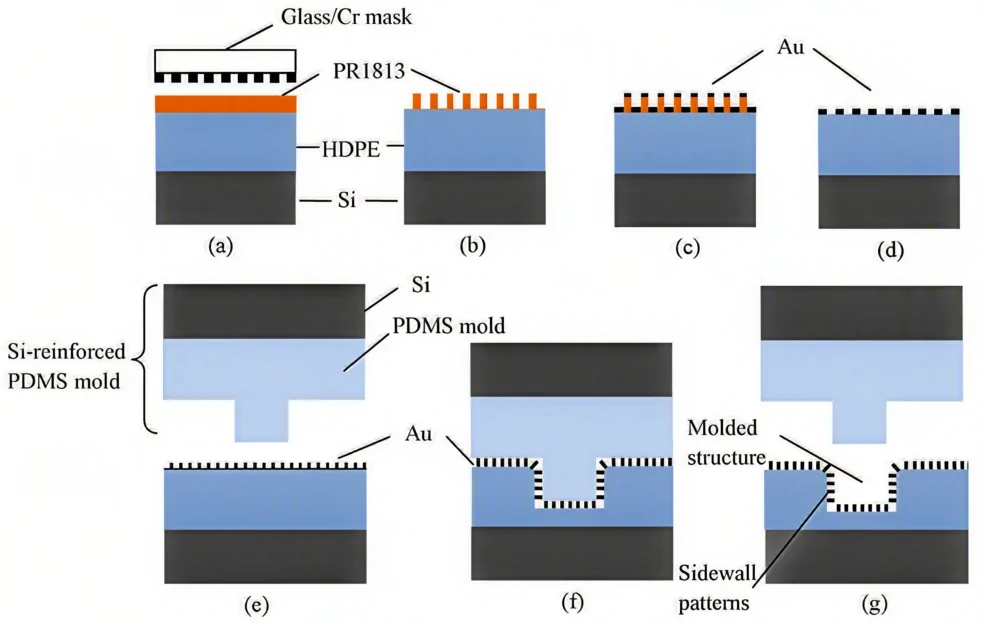
Schematic of the process used to fabricate Au sidewall patterns on polymer (HDPE) microstructures [9]. (**a**) UV lithography mask alignment on a photoresist-coated HDPE sheet; (**b**) photoresist patterning; (**c**) thermal evaporation of a gold film; (**d**) lift-off/removal of photoresist leaving Au top patterns; (**e**) heating the HDPE sheet slightly above its glass transition temperature; (**f**) hot-embossing contact using a Si-reinforced PDMS mold; and (**g**) mold separation leaving the transferred Au patterns on the sidewalls and bottom surfaces of the formed microstructures. Adapted from [9]. Permission obtained.

Within this manufacturing framework, the cutting operation employs rigid punch heads with sharp vertices and vertical edges. Driven by highly concentrated localized shear stresses, these tools rapidly sever polymeric substrates to yield discrete, decoupled micro-features on top horizontal surfaces. Conversely, addressing the vertical sidewall patterning bottleneck requires implementing the drawing operation modality. In this configuration, instead of severing the target material, the assembly deploys a specialized structural embossing mold featuring continuous, smooth rounded-corner geometries (Figure 4; [9]).

The fundamental mechanical and rheological workflow begins by pre-patterning high-fidelity metal micro-arrays onto the flat top surface of a thermoplastic substrate (Figure 4a,b). For example, conductive Au dot matrices or micro-lines can be defined on high-density polyethylene using standard planar processing or intermediate-layer transfer (Figure 4a–f; [9,39]). Subsequently, the entire system is heated above the polymer substrate’s glass transition temperature or melting temperature. This thermal transition softens the polymer into a visco-plastic state with enhanced fluidic compliance [43]. At this juncture, the rounded-corner punch head or a flexible composite mold is brought downward under a controlled mechanical load, vertically penetrating the softened polymer matrix (Figure 4e,f).

As the mold advances downward, implementing smooth, rounded contours replaces sharp shearing edges. This modified geometry efficiently redirects the unidirectional vertical load into a strong lateral shear stress field at the micro-interfaces [15,44]. This field drives the polymeric material to flow horizontally and climb upwards along the lateral sidewalls of the mold. Robust interfacial adhesion and mechanical interlocking develop between the softened polymer matrix and the metal thin film. The Au micropatterns originally residing along the top edges migrate in tandem with the spatial flow of the underlying polymer melt (Figure 4g).

The features are smoothly and continuously drawn and relocated onto the vertical sidewall surfaces of the newly generated microchannels. By precisely optimizing thermal profiles, embossing depths, and shear strain rates, this protocol circumvents the stress dead zones inherent to traditional imprinting. It enables a controlled transition from micro-cutting to tensile micro-drawing. Consequently, it yields high-resolution sidewall metallic arrays with intact pattern continuity [9,40].

### 3.2. Structural Optimization and Distortion Mitigation via Si-Reinforced PDMS Molds

Both all-metal hard punch dies and pure elastomer stamps can execute tensile drawing and transfer of sidewall designs during hot embossing. However, both individual material systems exhibit substantial mechanical deficiencies when scaled to micrometer or sub-micrometer alignment fidelities. Rigid metal molds frequently introduce mechanical scuffing, micro-scratches, or peeling fractures at sharp multi-planar corners due to thermal expansion coefficient mismatches during cyclic demolding. Conversely, purely flexible PDMS masters exhibit a low elastic modulus, typically scaling around 1–3 MPa. When subjected to vertical hot embossing forces, they inherently undergo substantial horizontal misalignment, elastic buckling, and asymmetrical bending deformations [32,45]. This structural instability of the soft master causes widespread geometric distortion of the resulting sidewall features, disabling precision-aligned micro-manufacturing.

To resolve this instability from a solid mechanics standpoint, a hybrid Si-reinforced PDMS mold fabrication protocol was engineered [9,46]. The core architecture relies on a multi-material hybrid design. A single-crystalline Si micro-skeleton with high rigidity (elastic modulus of 130–160 GPa) is embedded within a compliant, conformal elastomeric PDMS matrix [47]. The specific manufacturing sequence begins with reactive ion etching or deep reactive ion etching on a single-crystal Si wafer. This process fabricates high-density arrays of Si microfeatures with precise height control, typically on order of tens of micrometers. Following this step, high-temperature thermal oxidation grows an *in situ*, dense ${\mathrm{SiO}}_{2}$ reinforcement layer on the microstructured Si surface. This dielectric layer substantially increases the structural rigidity of the internal skeleton and optimizes local interfacial stress distribution. Finally, a liquid PDMS prepolymer is spin-coated and cast over this Si substrate bearing the ${\mathrm{SiO}}_{2}$-reinforced skeleton. Following thermal curing and systematic demolding, a high-fidelity composite mold is yielded, coupling an ultra-rigid internal Si backbone with the good elastic compliance of an external PDMS boundary layer [9].

During the sidewall patterning and hot-embossing sequence, this composite mold displays good mechanical performance [40]. When vertical thermal loads impinge on the hybrid stamp, the internal rigid Si/SiO_2_ skeleton absorbs the vast majority of compressive and bending stresses. This skeletal loading mechanism firmly locks the horizontal spatial coordinates of the mold features. Consequently, it compresses lateral deflections and asymmetrical deformations to a near-zero limit, eliminating the horizontal misalignments endemic to standard soft tooling [9]. Concurrently, the thin external skin of elastomeric PDMS retains its compliant surface mechanics, securing molecular-level conformal contact with the softened polymer substrate.

**Figure 5:**
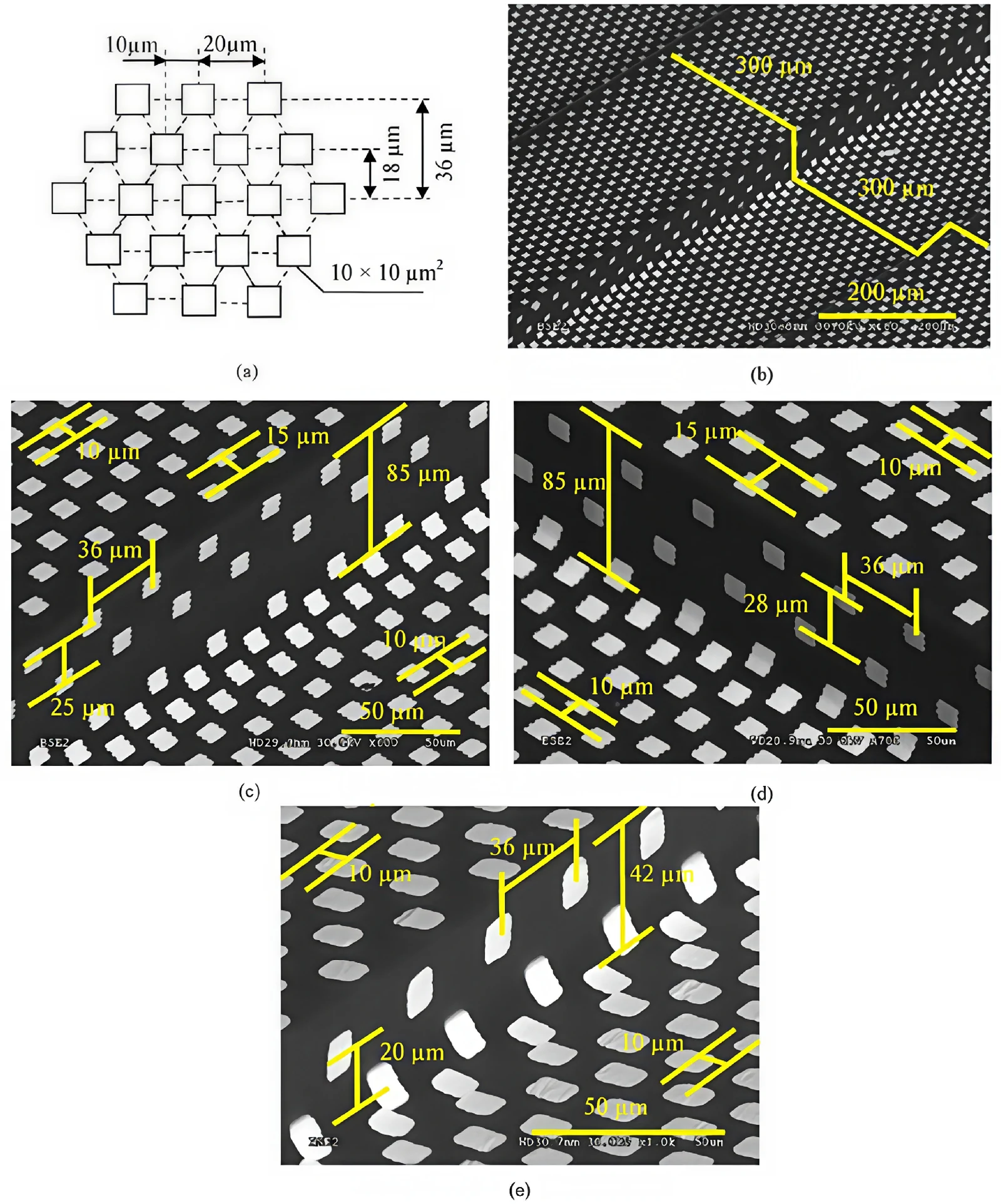
(**a**) Design of the Au dots on a flat surface. (**b**) The Au dots were relocated on top, side, and bottom surfaces of HDPE microchannels using the developed approach. The five lines show the profile of a microchannel. Close-up views of (**c**) left and (**d**) right sidewalls of a microchannel, indicating that two arrays of the Au dots were generated on either sidewall. (**e**) An array of Au dots was generated on the left sidewall of a 42-μm-deep microchannel [9]. Adapted from [9]. Permission obtained.

Utilizing this structurally optimized tooling, the distortion-free transfer and fabrication of single and dual arrays of Au dots across the 42μm and 85μm high vertical sidewalls of polymer microchannels were successfully demonstrated (Figure 5; [9]). The protocol demonstrated operational reliability by fabricating continuous Au interconnect wires—with a total width of 110μm—directly along these channel sidewall boundaries. This achievement marks a critical milestone for integrating functional electronics via mechanical flow relocation.

### 3.3. Fabrication and Multi-Dimensional Wetting Characterization of Integrated Multi-Surface Superhydrophobic Channels

**Figure 6:**
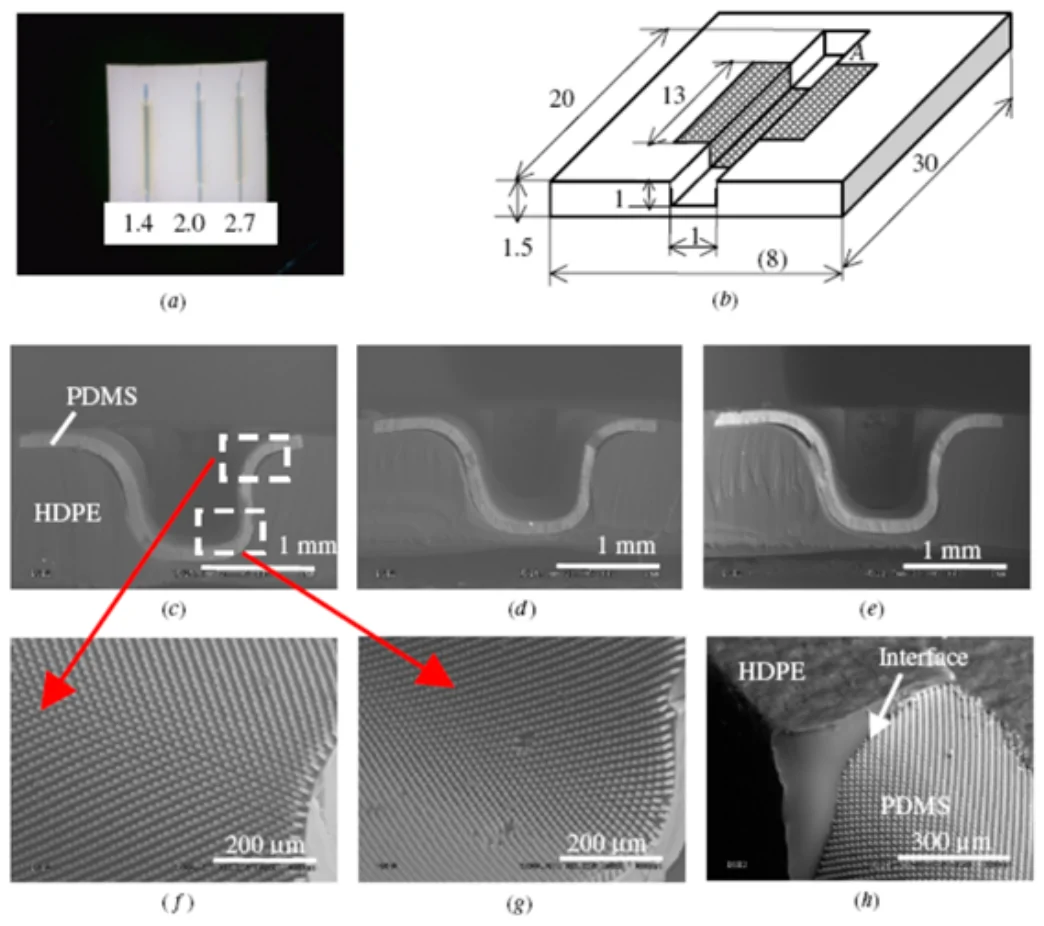
(**a**) 3D (optical) view of the three representative channels covered with PDMS pillars of aspect ratios of 1.4, 2.0 and 2.7, respectively; (**b**) dimensions of the designed super-hydrophobic channel (unit: mm); cross-section profiles of the channels covered with PDMS pillars of aspect ratios of (**c**) 2.7, (**d**) 2.0 and (**e**) 1.4; and close-up views of (**f**) the top corner, (**g**) the bottom corner and (**h**) the PDMS–HDPE interface at the 2.7 channel. The interface shown in (**h**) was labeled in (**b**) as “A” [15]. Adapted from [15]. Permission obtained.

**Figure 7:**
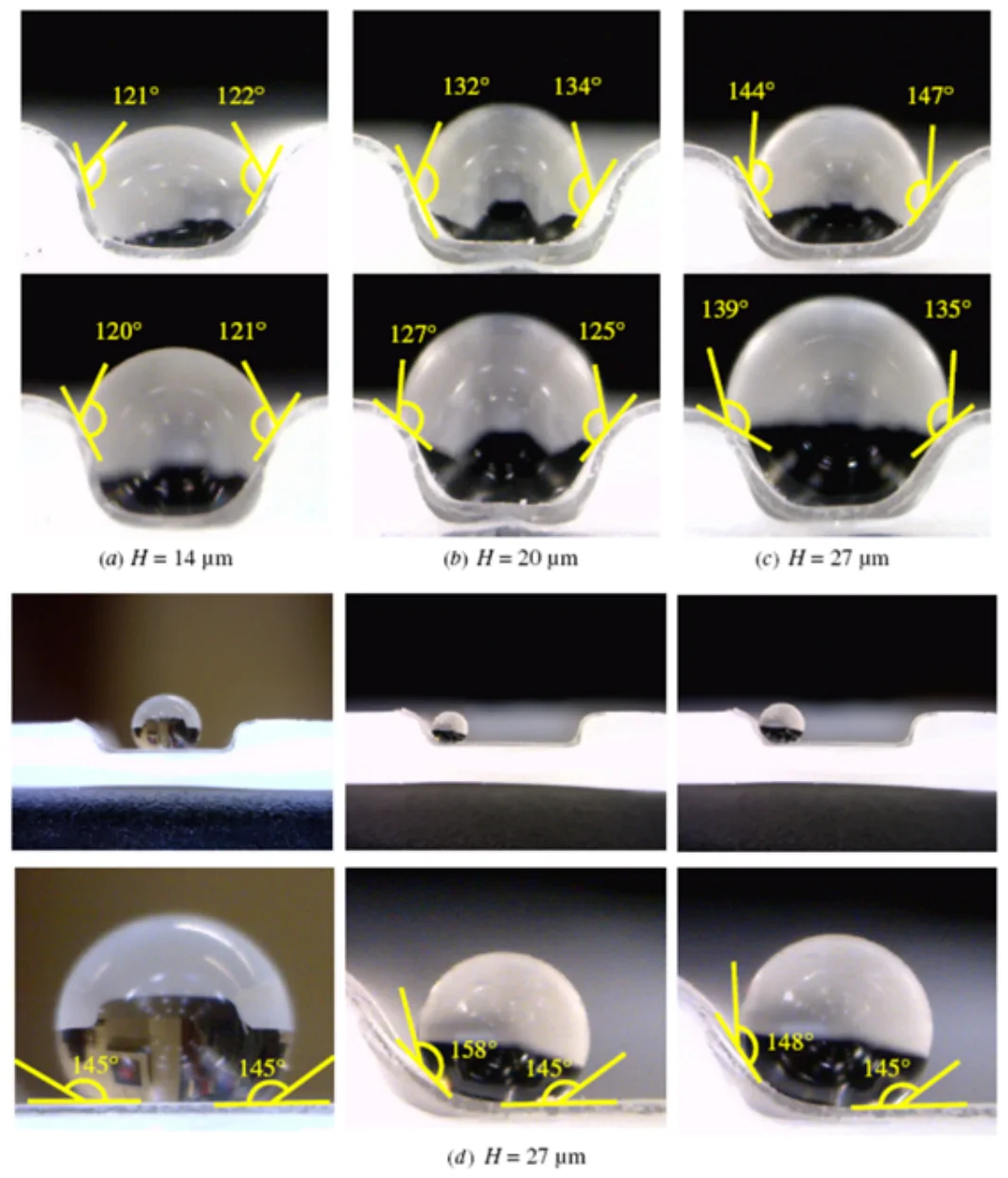
Contact angles of water at the middle and top spots of the sidewalls in the generated channels of width 1 mm and aspect ratios (**a**) 1.4, (**b**) 2.0 and (**c**) 2.7, respectively. (**d**) Contact angles of water at the bottom surfaces, and bottom and middle spots of the sidewall in a 4 mm wide channel covered with PDMS pillars of aspect ratio 2.7 [15]. Adapted from [15]. Permission obtained.

The technical utility of compliant hot-embossing transfer extends beyond multi-planar circuitry to reshaping the interfacial hydrodynamic functionalities of 3D microfluidic architectures. Conventional drag-reduction microchannels exhibit a structural asymmetry: topographical features are restricted to the bottom substrate, leaving laminar flows subject to high viscous shear stress along vertical sidewalls [15]. To eliminate this manufacturing blind spot, a multi-surface fabrication protocol was established. This approach deploys a thin, microstructured elastomeric film, such as a PDMS micropillar array, as a functional conformal skin to line rigid mold cavities, followed by hot embossing of a thermoplastic polymer matrix [15]. By systematically tuning the feature aspect ratios from 1.4 up to a mechanical limit of 2.7 and center-to-center pitches from 15–24μm, this framework secured unbroken topological coverage across both the channel floor and dual vertical sidewalls (Figure 6). This continuous coating pushed the local water contact angles up to 158°, promoting low-drag slip boundary conditions that sustain a gas plastron within moving streams (Figure 7; [15]).

This multi-surface drag-reduction strategy may be indispensable in the domain of biomedical microdevices. Within microscale high-throughput inertial microfluidic sorting chips, viscous shear resistance from conventional unpatterned vertical sidewalls heavily distorts the parabolic velocity profile. This flow distortion disrupts optimal inertial focusing and compromises cell separation trajectories [48]. As noted by Di Carlo (2009) [48], extending low-drag slip microstructures from the channel floor to the vertical sidewalls can significantly reduce hydraulic resistance in deep microchannels by increasing the effective slip boundary over the entire channel perimeter.

### 3.4. Micro-Transfer Printing

**Figure 8:**
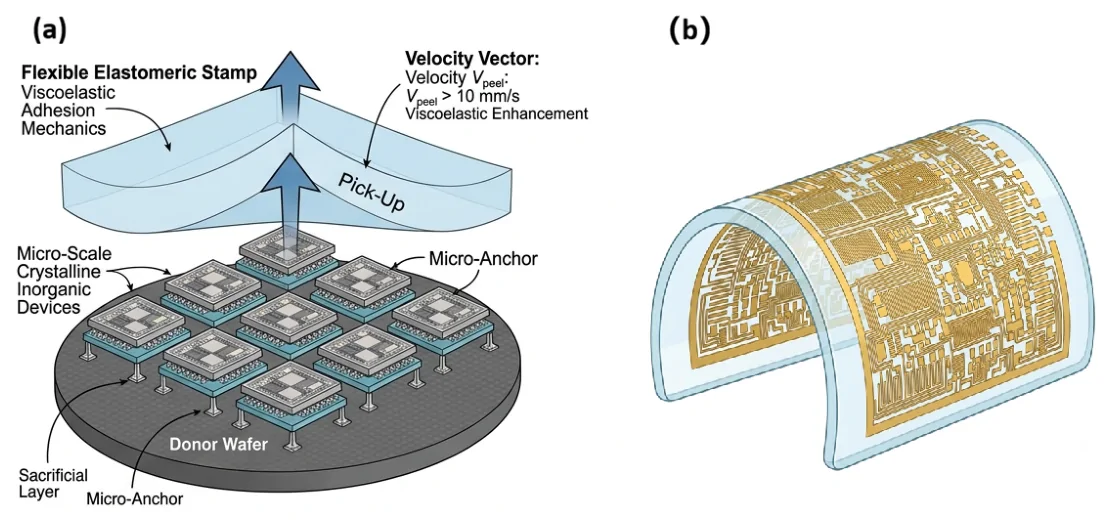
Schematic of viscoelastic micro-transfer printing pathways for planar pickup and non-planar assembly. (**a**) Planar retrieval phase: rapid peeling of an elastomeric stamp induces rate-dependent viscoelastic enhancement of the interfacial fracture energy, fracturing micro-anchors to pick up micro-device arrays from a sacrificial donor wafer. (**b**) Non-planar transfer phase: slow peeling enables stress relaxation, releasing micro-scale functional patterns conformally onto curved three-dimensional receiver surfaces, such as cylindrical optical substrates.

Micro-transfer printing may also be used for pattern relocation on cylindrical substrates. As shown in Figure 8, in this approach, microstructures or devices could be transferred from a donor surface to a target surface using a flexible elastomeric stamp [4,49,50,51]. During the transfer phase, the architecture implements an advanced speed-dependent viscoelastic adhesion mechanism [52]. When an elastomeric stamp contacts the source wafer and is rapidly peeled away, which is typically at velocities around 1mm/s, strong kinetic effects come into play. The rate-dependent viscoelasticity of the polymer ramps up the effective interfacial fracture energy, enabling the stamp to successfully pick up the planar thin-film components (Figure 8a). Subsequently, the flexible stamp is wrapped around a cylindrical substrate to transfer the microstructures or devices onto its surface (Figure 8b; [49]).

The micro-transfer method has significantly expanded material compatibility beyond polymeric materials to include high-performance crystalline inorganic semiconductors, thereby providing a versatile platform for integrating functional electronic components onto three-dimensional surfaces. Unlike hot-embossing techniques, which are generally limited to thermoplastic substrates, this method can be applied to both rigid and flexible substrates, enabling the fabrication of smart electronic skins on a wider range of 3D structures. However, as illustrated in Figure 6, the folded PDMS membrane forms rounded corners during deformation and struggles to achieve full conformal contact with sharp 90° corners. Consequently, the finite bending radius of the elastomeric stamp makes it difficult to achieve intimate conformal contact with vertical channel sidewalls, especially in deep or high-aspect-ratio structures. As a result, this approach is more suitable for patterning inclined or smoothly curved surfaces, such as cylindrical substrates, than vertical sidewalls featuring sharp corners.

## 4. Pattern Relocation via Strain Recovery of Thermo-Responsive Shape Memory Polymer

### 4.1. Mechanical Mechanism and Technological Evolution of the Dual Strain-Recovery Architecture

Although sidewall patterning in Section 3 was successfully executed using the drawing operation of the MPL, the underlying process heavily relied on external compressive loads and rigorous shear-rate controls from physical molds. Stimulus-responsive polymeric substrates are highly susceptible to thermal reflow, softening, or structural degradation at elevated temperatures. Consequently, intensive mechanical stamping frequently triggers the geometric collapse of delicate 3D microstructures on these sensitive materials. To liberate the process from dependency on external rigid tooling, a self-driven sidewall patterning lineage was developed, mediated by the massive strain recovery of thermo-responsive shape memory polymers [15,53].

**Figure 9:**
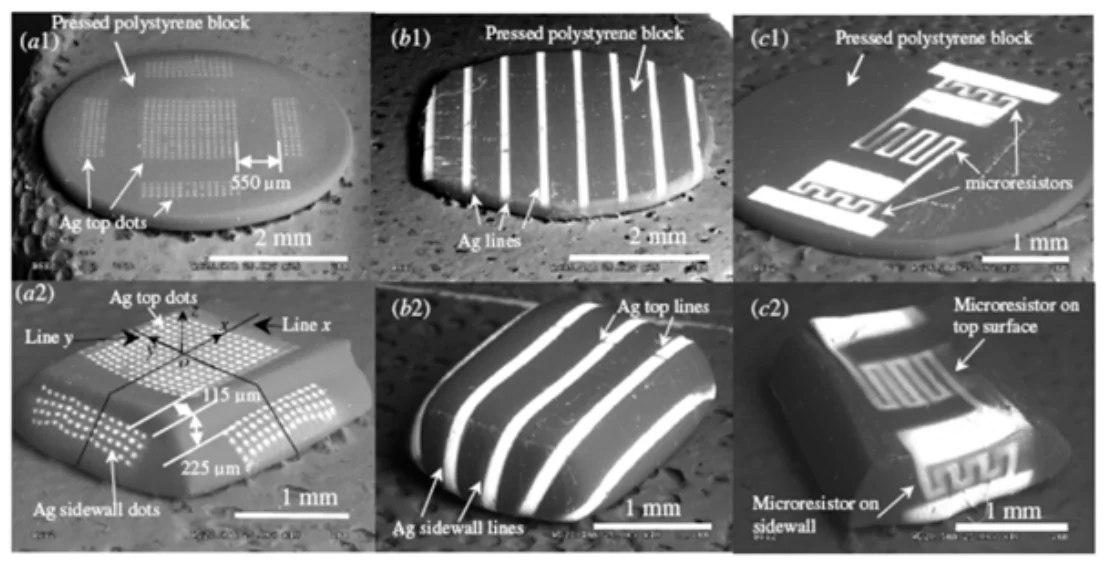
(**a1**) $50\times 50\;\mu m^{2}$ Ag dots, (**b1**) 150 μm wide Ag lines, and (**c1**) serpentine-shaped microresistors were generated on the top surfaces of pressed polystyrene blocks using a stencil technique. (**a2**) The dots, (**b2**) lines and (**c2**) microresistors were generated on both top and side surfaces of the polystyrene blocks after strain recovery at 160 °C [17]. Adapted from [17]. Permission obtained.

The core responsive substrate relies on commercially biaxially pre-stretched polystyrene (PS) blocks, which store a massive tensile strain (scaling from 400% to 2000%) frozen within the extended polymer lattice at room temperature. To convert this unidirectional contraction mechanics into a self-contained driving force for relocating patterns vertically, our group developed a “Dual Strain-Recovery” method [17]. The workflow initially subjects the raw PS block to unconstrained thermal relaxation at 160 °C to remove manufacturing strains. Subsequently, it hot-embosses a temporary trench topology with sharp vertices to freeze a localized stress field, followed by pre-depositing planar metallic designs (e.g., conductive Ag matrices) along the flat horizons. Upon a second unconstrained thermal activation at 160 °C without rigid mold clamping constraints, the substrate sweeps past its glass transition temperature ($T_{g}\approx 100\;{}^{\circ}C$). This thermal activation abruptly releases the highly concentrated tensile strain field localized around the trench edges. Consequently, it forces the channel sidewalls to undergo sharp inward shrinkage while the channel depth is forcibly restored upward. Driven by this contraction-restoration lattice motion, the Ag micropatterns originally residing along the edge of the flat horizon are rotated and relocated onto the finalized vertical sidewall surfaces (Figure 9; [17]).

**Figure 10:**
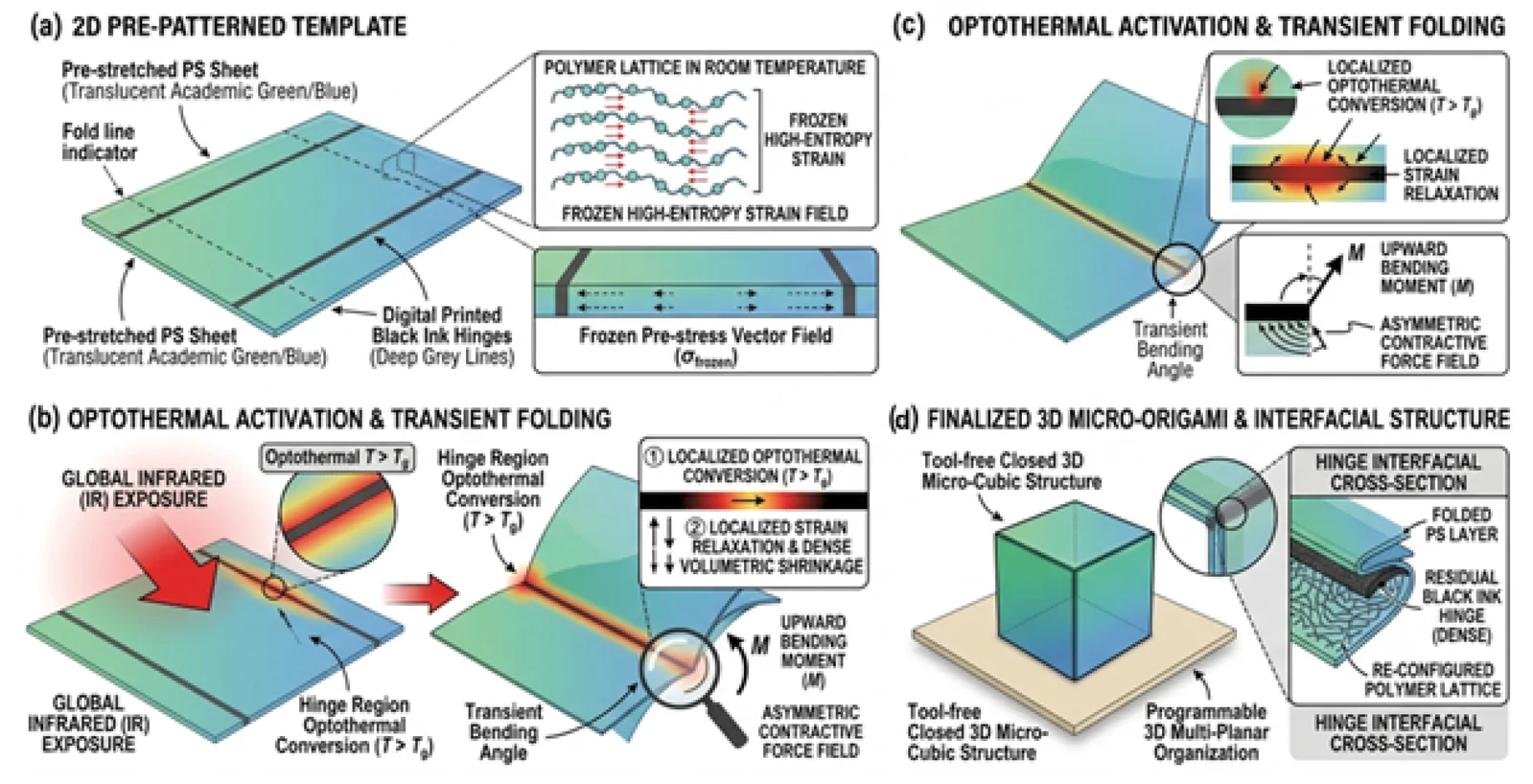
Schematic illustration of spatially localized optothermal self-folding dynamics of pre-stretched polymer sheets. (**a**) 2D patterned template: selective deposition of light-absorbing black ink hinges onto a biaxially pre-stretched shape memory substrate that stores high-entropy internal frozen strains ($\sigma_{\mathrm{frozen}}$). (**b**) Optothermal activation: under global infrared radiation, highly localized optothermal conversion exclusively at the ink-marked lines elevates the local temperature beyond $T_{g}\approx 100\;{}^{\circ}C$. (**c**) Localized strain-recovery folding: this sharp thermal activation abruptly releases the local frozen tensile strain field, generating a robust asymmetric bending moment across the hinge zone to drive spontaneous out-of-plane folding. (**d**) Finalized 3D micro-origami: complete evolution into a closed 3D cubic structure tool-free, establishing a programmable paradigm for moldless multi-planar device organization. (Adapted from [54]). Adapted from [54]. Permission obtained.

Because uniform heating (e.g., in a conventional oven) triggers simultaneous contraction across the entire polymeric substrate, spatial pattern flexibility remains inherently constrained. To overcome this limitation, other researchers introduced localized photothermal activation to this mechanism [54]. This strategy inkjet-prints light-absorbing ink traces onto pre-stretched polymer sheets, followed by global infrared or laser illumination. This exposure selectively triggers localized strain recovery, enabling programmable 3D folding. This configuration ensures that optical energy undergoes highly efficient, spatially localized optothermal conversion exclusively at the ink-marked regions. Consequently, the local strain field is selectively released under low ambient temperatures, driving the thin film to spontaneously undergo complex, asymmetric self-folding. This spatial control elevates the programmability of 3D sidewall patterning (Figure 10; [17,54]).

Concurrently with the rapid growth of wearable healthcare technologies and soft robotics, this mold-free, self-driven deformation route enabled the scalable production of high-performance, complex functional architectures. Research has expanded far beyond the mere transfer of isolated metallic dot matrices by integrating this strain-recovery mechanism into composite shape memory polymer networks. These responsive platforms react to multiple orthogonal stimuli, including light, electricity, magnetism, and humidity. Consequently, multi-layered single-crystalline Si sensors and nanowire OLEDs can be conformally wrapped onto high-curvature biomimetic 3D surfaces—such as artificial retinal-shaped micro-hemispherical shells and soft robotic gripper flaps—without requiring post-fabrication mechanical alignment [55,56]. This strategy enables non-planar electronic skins with tactile and environmental sensing capabilities, transitioning micro-fabrication from traditional planar surfaces to integrated, multi-functional 3D systems [55,56].

### 4.2. Mitigation of Edge Fractures, Surface Roughness Reflow, and Micro-Resistor Performance

To eradicate corner electrical open-circuits and optimize the continuity of sidewall devices, our group systematically investigated the thermal reflow mechanism of the polymer above its transition node. Specifically, this surface roughness evolution was governed by tuning boundary conditions during the second strain recovery phase [17]. Under a long-duration thermal soak at 160 °C, the polystyrene (PS) substrate enters a softened state driven by the thermodynamic minimization of surface energy. Consequently, the high-frequency microscopic roughness undergoes localized micro-reflow and structural reorganization. This microscopic reflow effect delivers a critical mechanical advantage: it forces the originally sharp, rigid 90° step edges to spontaneously blend into a smooth, continuous, low-strain bending profile. Introducing rounded geometries effectively alleviates stress concentration at multi-planar vertices. This smooth transition allows metallic Ag circuit wires to cross from top flat surfaces onto vertical sidewalls without cracking or circuit failure [17].

However, thermal relaxation contraction inevitably introduces a microscopic destabilization at the metal–polymer bilayer interface known as the micro-wrinkling effect [57]. A massive Young’s modulus mismatch exists between the inorganic metallic Ag thin film (high elastic modulus) and the rubbery soft PS matrix (low elastic modulus). Consequently, as the substrate contracts at 160 °C, the Ag thin film undergoes spontaneous, localized elastic buckling to generate periodic 3D micro/nanoscale wrinkles [57,58,59]. While the development of these micro-wrinkles sacrifices macroscopic flat topography by increasing surface roughness, it provides irreplaceable solid-state physics utility. The wrinkled network functions as a spring-like “geometric stretchable buffer” within the metallic lattice, absorbing and dissipating intensive internal strain energy through the unfolding and folding of its wave crests. This dynamic buffer mechanism successfully blocks the nucleation and propagation of fatal macroscopic fractures during major spatial relocations [17,60].

**Figure 11:**
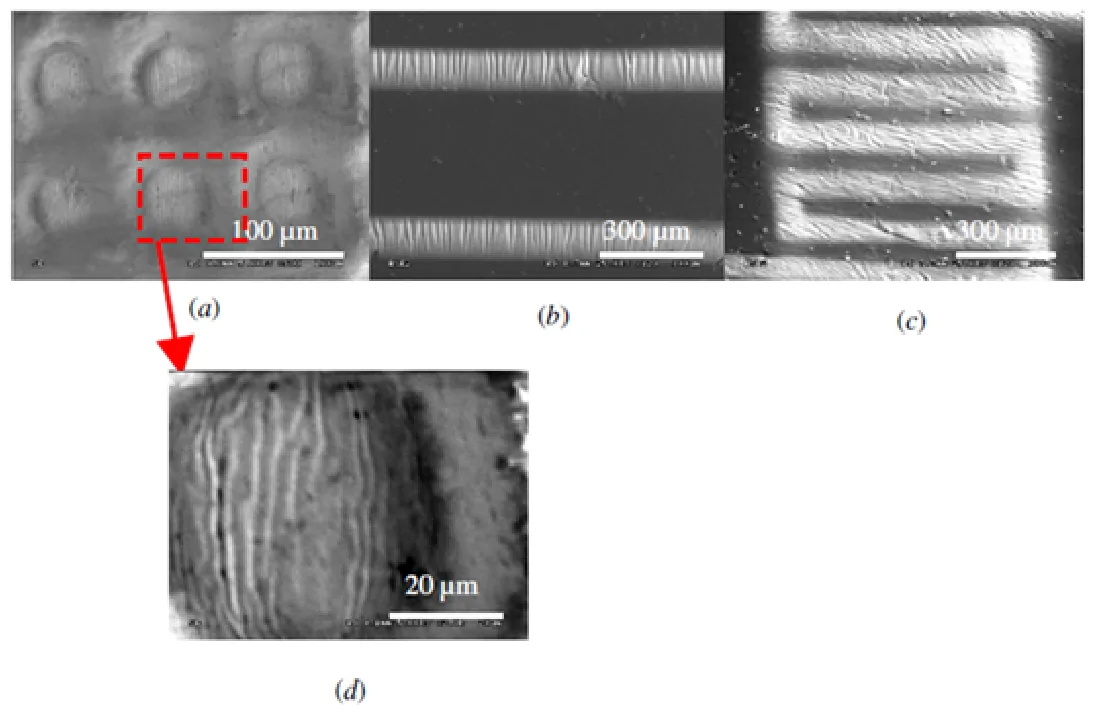
Interfacial wrinkle and buckling topologies of Au thin films on thermally contracted PS matrices. (**a**–**c**) Morphological evaluations of fabricated microstructures exhibiting strain-induced sinusoidal wrinkling on the top planar surfaces of compressed polymer blocks: (**a**) isolated dot configurations, (**b**) continuous conductive microlines, and (**c**) microheater grid networks. (**d**) High-magnification close-up micrograph tracking the localized buckle profile and periodic wavelength of the wrinkled dot array in (**a**) [17]. Adapted from [17]. Permission obtained.

To quantitatively evaluate the electrical utility of this self-driven sidewall patterning approach for 3D electronics, our group fabricated continuous 3D micro-resistors directly across the finalized vertical sidewalls. Subsequently, we executed rigorous four-probe electrical interconnect and resistance characterizations on these structures [17]. Micro-wrinkled topographies slightly extend the effective conductive path along the 3D surface. As a result, the electrical resistance of the sidewall micro-resistors scales up marginally relative to flat planar theoretical predictions, remaining well within controlled bounds. Multi-point cyclic resistance characterizations across batch samples verified that the sidewall micro-resistors maintained stable Ohmic continuity, free of open-circuit failures induced by corner tearing. This outcome demonstrates the mechanical feasibility and industrial reliability of applying the shape-memory strain recovery to construct complex 3D sidewall circuits and electronic skins in situ with high fidelity. Specifically, this approach bypasses the need for external physical compression tooling (Figure 11; [17]).

### 4.3. Hinge-Based Self-Folding Methods

**Figure 12:**
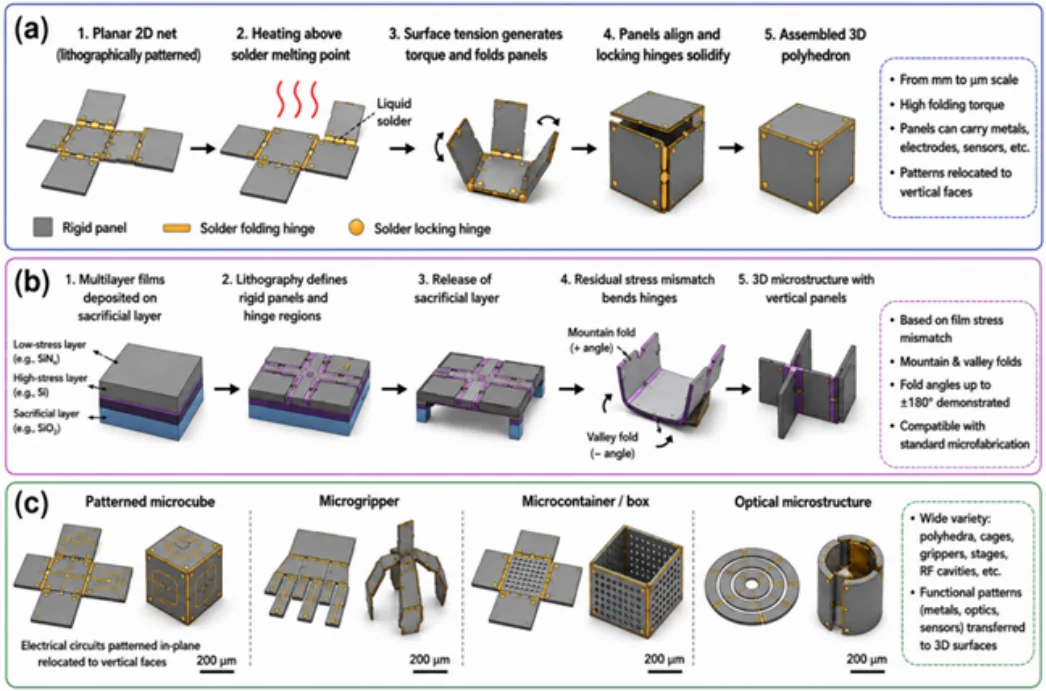
(**a**) Surface-tension (solder) hinges: 2D net to 3D polyhedron. (**b**) Residual-stress-driven hinges: micro-origami. (**c**) Examples of functional structures obtained by hinge-based self-folding [54,61,62,63].

Hinge-based self-folding provides another route for relocating planar micro/nanopatterns onto three-dimensional surfaces [61]. In this approach, rigid or semi-rigid panels are first patterned in a planar configuration and interconnected by deformable or active hinges. Upon activation by surface tension, residual stress, thermal deformation, swelling, or other stimuli, bending moments generated at the hinges rotate the panels out of plane and assemble them into prescribed 3D geometries [54,61,62]. Because functional structures can be defined on the panels prior to folding using conventional planar lithography, the subsequent self-folding process effectively relocates these patterns onto vertical, inclined, or enclosed sidewalls [61,63]. Surface-tension-driven solder hinges have enabled self-assembled, arbitrarily patterned metallic polyhedra with dimensions ranging from approximately 2 mm to 15 μm (Figure 12a; [62]), while stress-driven self-folding approaches (Figure 12b) further extend this concept toward micro- and nano-origami structures and functional 3D devices (Figure 12c; [63]).

Hinge-based self-folding methods offer a more controllable approach than the local strain-recovery of shape memory polymers demonstrated by Liu and co-workers (2010) [17], as the latter may have difficulty forming precisely defined vertical sidewalls. In addition, hinge-based self-folding provides greater flexibility in substrate material selection, since it is not restricted to shape memory polymers.

## 5. Flexible Membrane-Mediated Stenciling and Directional Shadow Masking

### 5.1. Patterning Bottlenecks on Rigid Inorganic Interfaces and Hexane-Assisted Through-Hole Membrane Fabrication

The mechanical drawing, hot embossing, and shape-memory-driven transformations detailed in Section 3 and Section 4 successfully achieved sidewall patterning. However, these technical lineages share a material prerequisite: the substrate itself must be a deformable polymer capable of undergoing intensive viscoelastic flow or elastic strain recovery. However, for the rigid inorganic substrates driving the semiconductor microchip industry, such as single-crystalline Si or quartz glass, the corresponding boundaries possess massive Young’s moduli typically scaling from 130–160GPa. These displacement and shrinkage routes are limited by their inability to accommodate large deformations. This challenge becomes acute when confronted with rigid vertical Si boundaries, such as vertical 110 crystal planes or discrete trenches accurately exposed via anisotropic wet etching. Under such rigid constraints, forcing pattern attachment via physical compressive molding is mechanically impossible [64].

To clear this manufacturing blind spot on inorganic rigid interfaces, an alternative pathway was engineered: a non-destructive 3D stenciling lineage utilized free-standing, ultra-thin through-hole PDMS membranes as flexible shadow masks [18]. To achieve high-fidelity, high-resolution shadow stenciling, the flexible PDMS membrane must possess a geometric configuration of completely open, residue-free through-holes. Within conventional elastomeric micromachining, directly spin-coating ultra-thin films across rigid microscale master molds inherently leaves a residual layer, or scum, over the top horizons of the mold structures. The residual thickness, which ranges from several hundred nanometers to micrometers, is driven by fluid surface tension and capillary pinning. This microscopic residual layer blocks the windows, rendering shadow mask pattern transfer entirely non-functional [65]. In addition, conventional reactive ion etch used to strip the scum compromises the structural integrity of the ultra-thin skin, crippling throughput yields during large-scale operations.

**Figure 13:**
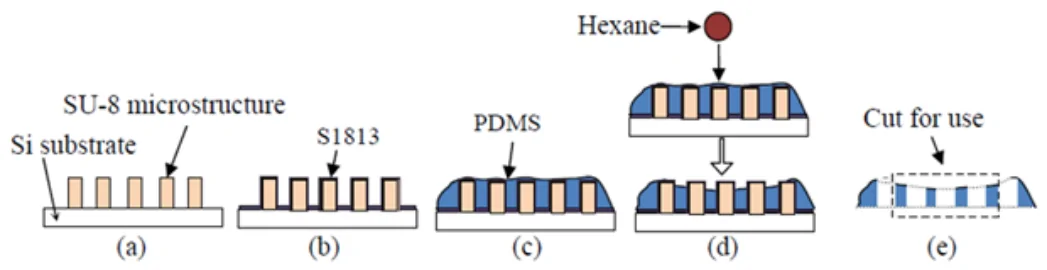
Fabrication of a PDMS membrane with hollow structures: (**a**) generate SU-8 microstructures; (**b**) spin-coat a positive photoresist S1813; (**c**) spin-coat a PDMS solution; (**d**) apply liquid hexane to remove the PDMS from the top surfaces of the SU-8 microstructures; and (**e**) cure the PDMS, remove the PDMS membrane from the substrate, and cut off the central area of this membrane for later use [18]. Adapted from [18]. Permission obtained.

To resolve this material-shaping bottleneck, a specific process was developed to fabricate the hollow PDMS mask (Figure 13; [18]). Initially, a high-density array of microscale pillars or ridges is engineered across a standard Si wafer to serve as a rigid male mold for spatial through-hole coordinates. This structural template is fabricated using SU-8, a high-aspect-ratio negative photoresist. To release the PDMS film from the template at the end of the process, positive photoresist S1813 is first spin-coated over the template. Subsequently, a precisely measured volume of uncured liquid PDMS prepolymer is spin-coated over this micro-structured platform. Driven by surface fluidic dynamics, the liquid “PDMS” undergoes localized splitting and climbing over the top surfaces of the “SU-8” pillars, generating the viscous blocking scum (Figure 13c).

During the critical temporal window before thermal curing, a high-speed spin-rinsing sequence is introduced. It utilizes a low-surface-tension organic solvent, liquid hexane. Driven by high-speed centrifugation, a liquid hexane stream exerts gentle fluidic shear while exploiting its high chemical solubility toward uncured PDMS. This action precisely flushes away residual PDMS from the SU-8 pillar tops without disturbing the polymer layer deposited inside the trench cavities [18]. Following thermal curing, the photoresist sacrificial anchors are dissolved using acetone. This release step enables the flexible shadow mask to be successfully molded and peeled away. This mask adopts a specialized thin-center, thick-edge multi-scale architecture. Specifically, its central window domain constitutes an ultra-thin (≤10 μm) open-aperture high-precision through-hole membrane, whereas its peripheral boundary maintains a thickened structural handle with a thickness scaling in the hundreds of micrometers (Figure 13e).

### 5.2. Conformal Wrapping Sidewall Patterning

The unique “thin center, thick edge” multi-scale architecture delivered by the hexane-assisted protocol ensures low bending rigidity in the central domain, while endowing the outer stencil with high mechanical tensile strength. This structural balance prevents elastic tearing or wrinkling during manual demolding and alignment tensioning. When aligned over a high-aspect-ratio rigid Si trench, the thin central membrane adaptively bends and self-wraps tightly along the vertical sidewalls, driven spontaneously by the evaporation of an intermediate solvent, such as isopropanol, via interfacial surface tension and capillary forces [18,66]. Consequently, this secures conformal contact across the rigid inorganic boundary without requiring high-temperature hot embossing or risking substrate cracking.

**Figure 14:**
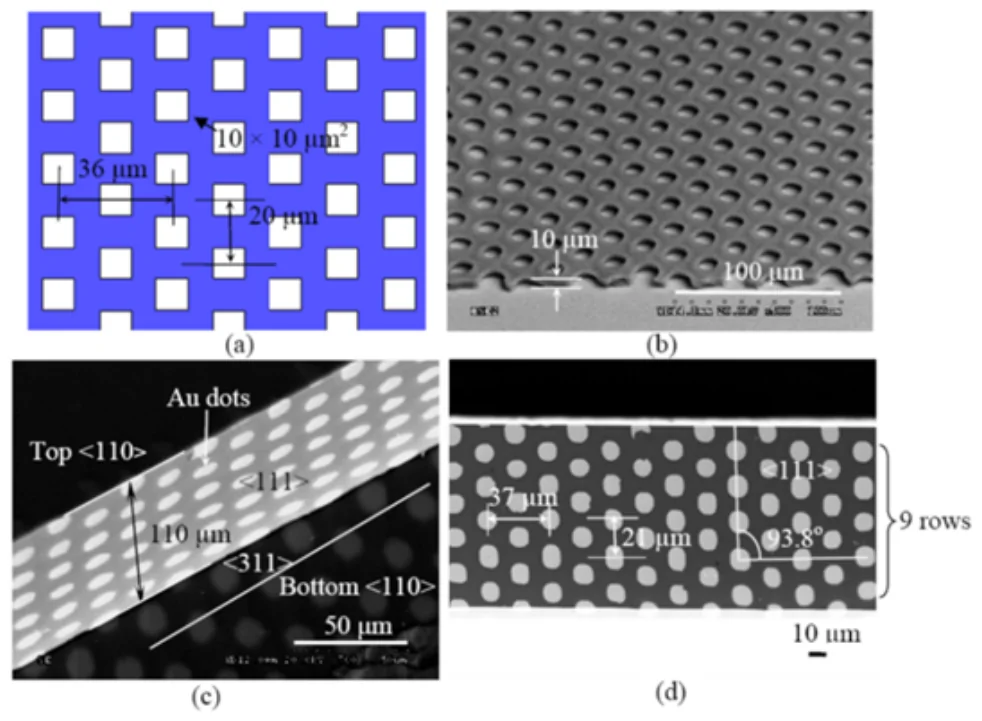
(**a**) Designed dot patterns on the mask; (**b**) fabricated PDMS membrane with 10μm through-holes; (**c**) 3D and (**d**) side views of 10μm Au dots generated on a vertical Si sidewall (i.e., {111} surface) [19]. Adapted from [18]. Permission obtained.

Subsequently, this compliant wrapping mechanism was expanded to handle more intricate inorganic 3D geometries and multi-surface topologies. Symmetrical multi-surface integration on single-crystalline Si requires simultaneous sidewall processing. By evolving the flexible shadow mask to conformally wrap a Si vertical sidewall Au micropatterns are generated on the vertical sidewall with high fidelity via sputtering (Figure 14; [19,67]).

### 5.3. 3D Projection Models and Tilted Evaporation

Because shadow stenciling cannot execute *in situ* chemical processing along vertical walls, its pattern transfer depends on the straight-line ballistic transport path of evaporated atoms. In traditional semiconductor manufacturing, normal-incidence vaporization generates a net-zero geometric projection area on vertical boundaries. To break this spatial constraint, an anisotropic Si trench wafer conformally wrapped with the through-hole flexible PDMS stencil was mounted onto a custom multi-axis tilting holder. This customized configuration enabled a precise, tilted directional thermal evaporation process across the microstructures. By tilting the substrate at a defined inclination angle θ, the geometric shadow effect cast by the microchannel’s top rim is employed to secure high-fidelity directional functionalization across rigid walls.

**Figure 15:**
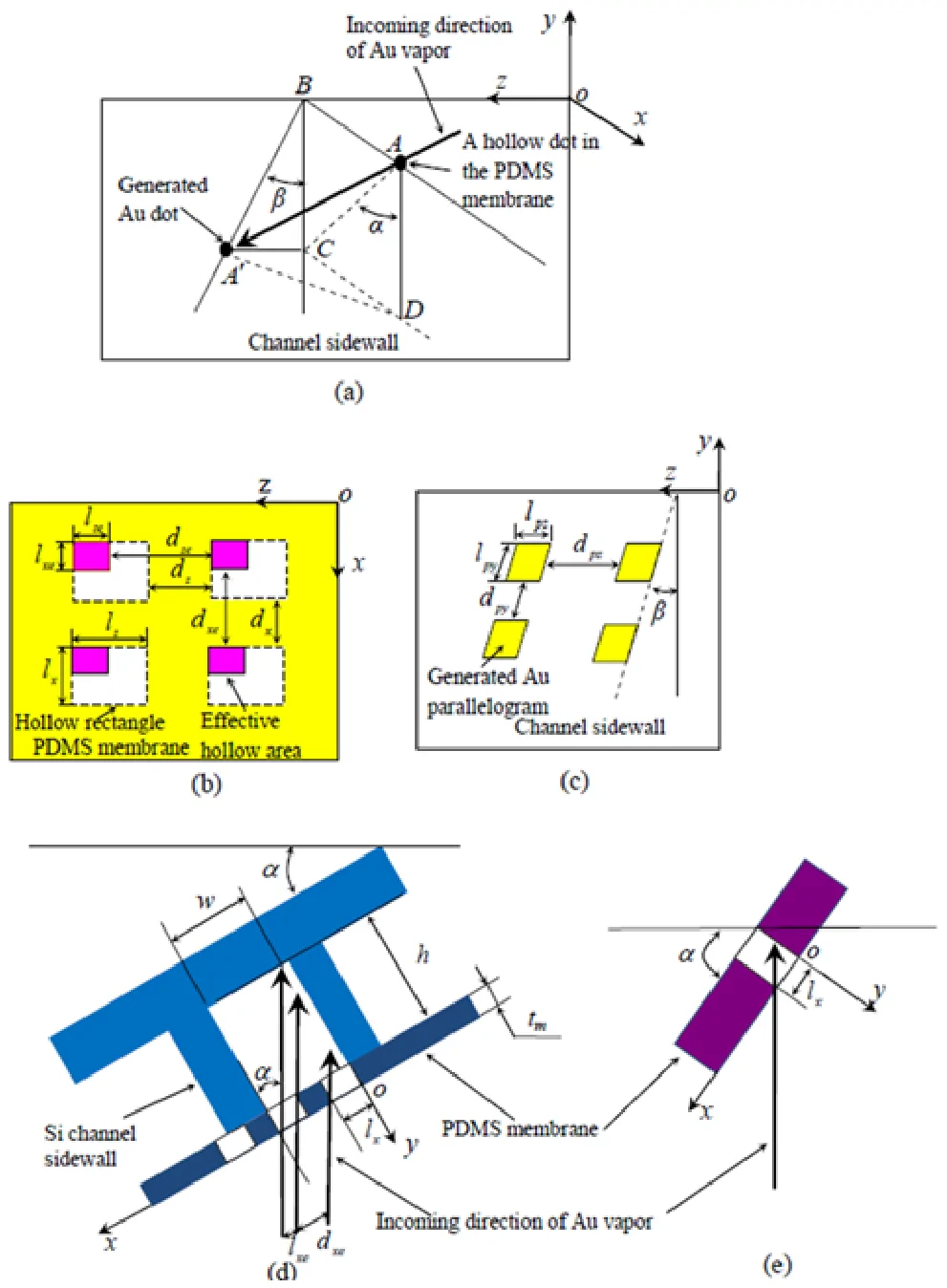
(**a**) 3D illustration of generating a Au dot on a channel sidewall. 2D illustrations of (**b**) masking structures and (**c**) generated Au patterns. (**d**) Side view of Au vapor penetrating a hollow structure in the PDMS membrane. (**e**) Side view of a case where Au vapor cannot penetrate a hollow structure of the PDMS membrane [19]. Adapted from [19]. Permission obtained.

To guide precision positioning, a 3D geometric projection model was formulated specifically for this tilted evaporation configuration [19,68]. Within a rectangular coordinate system (channel width *W*, depth *H*, mask hole diameter *d*, and pitch *P*), the ballistic kinetic trajectory of atoms is defined by an incident unit vector u(α,β), where α and β represent the inclination and azimuthal orientation angles, respectively (Figure 15). Due to the oblique trajectory, the resulting metallic designs undergo asymmetrical stretching. Additionally, because conventional scanning electron microscope (SEM) imaging lines of sight form a fixed oblique perspective, the direct 2D readings represent an elongated pseudo-projection. To reconstruct the true physical boundaries, our group integrated a coordinate transformation [19]:

(3) $$\begin{array}{cc} l_{\mathrm{pz}} & =l_{\mathrm{ze}}, \end{array}$$

(4) $$\begin{array}{cc} l_{\mathrm{py}} & =(l_{x}-t_{m}\tan\alpha )\cot\alpha \sec\beta , \end{array}$$

(5) $$\begin{array}{cc} d_{\mathrm{pz}} & =d_{\mathrm{ze}}, \end{array}$$

(6) $$\begin{array}{cc} d_{\mathrm{py}} & =(d_{x}+t_{m}\tan\alpha )\cot\alpha \sec\beta , \end{array}$$

where $l_{\mathrm{pz}}$ denotes the length of the top and bottom edges of a parallelogram (Figure 15), $l_{\mathrm{ze}}$ the dimension of an effective hollow area along the *z*-axis, $l_{\mathrm{py}}$ the length of the two side edges of the parallelogram, $l_{x}$ the dimensions of a hollow rectangle along the *x*-axis, $t_{m}$ the thickness of the PDMS membrane, $d_{\mathrm{pz}}$ the distance between two neighboring parallelograms along the *z*-axis, $d_{\mathrm{ze}}$ the distances between two neighboring effective hollow areas along the *z*-axis, $d_{\mathrm{py}}$ the distance between two neighboring parallelograms along the direction of the side edge of a parallelogram, and $d_{x}$ the distances between two neighboring hollow rectangles along the *x*-axis.

This geometric derivation verified the spatial mapping laws during tilted evaporation. Specifically, the top and bottom lengths of each deposited parallelogram remain identical to their physical counterparts in the effective hollow aperture. Invariant spatial scaling also governs the pitch distance between neighboring parallelograms along the vertical *z*-direction. In stark contrast, the oblique side boundaries undergo a significant dimensional transformation. These oblique edges contract or expand by a geometric ratio of cotαsecβ relative to their initial target baselines within the defining aperture.

Guided by this quantitative model, we developed a dual-step tilted evaporation process. This strategy enabled the simultaneous fabrication of dense Au dots and continuous Au microwires across both vertical sidewalls of deep Si trenches [18,19]. Empirical characterizations verified that the conductive microwires maintained good thickness uniformity and sharp boundary definitions across all 3D surface horizons. Specifically, high structural integrity was preserved across the horizontal top surfaces, the anisotropically exposed vertical {111} Si sidewalls, and the recessed bottom channel surfaces, supporting the mathematical determinism of the established model.

Other researchers also adapted this oblique-angle projection strategy to expand fabrication capabilities for nanowire gas sensors. Gao et al. (2011) [69] exploited the geometric shadowing associated with angled physical vapor deposition, in combination with nanoimprint lithography, to define silicon nanowires with controllable widths for highly sensitive ${\mathrm{NO}}_{2}$ gas sensing. They fabricated 60- and 90-nm Si nanowire gas sensors and report ${\mathrm{NO}}_{2}$ sensitivities of 155% and 44%, respectively [69].

## 6. Compliance-Driven Membrane Transfer, Contact-Mediated Interfacing, and Capillary Direct-Writing

### 6.1. Geometric Manufacturing Challenges and Compliance Mechanics on Non-Developable Surfaces

The manufacturing approaches detailed in the preceding sections encompass polymer deformation (Section 3), strain recovery (Section 4), and thin elastomeric stenciling (Section 5). Collectively, these approaches enable effective sidewall patterning on rectilinear or cylindrical structures. However, patterning irregular and geometrically complex surfaces remains challenging, particularly for structures such as the outer surfaces of glass micropipettes with curved tips.

**Figure 16:**
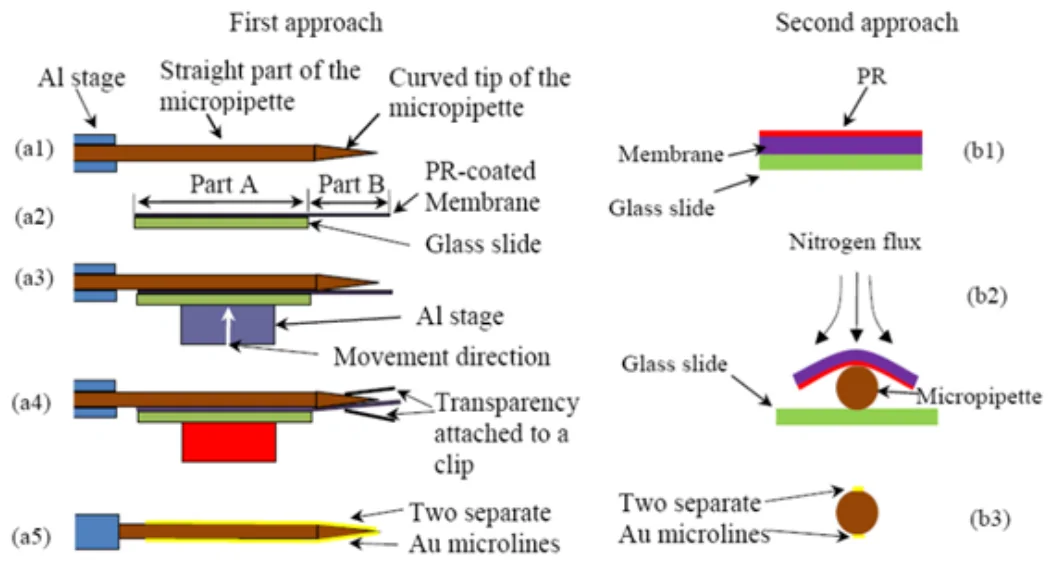
Schematics of fabrication procedures of the two approaches (not to scale). The first approach (top views): (**a1**) Deposit 100-nm-thick Au on the micropipette, and fix it between two Al stages, (**a2**) attach a membrane to a glass slide and spin-coat PR on the membrane, (**a3**) push Part A of the PR-coated membrane against the micropipette, transferring PR to the micropipette at the contact place (a PR line is formed on the straight tube of the micropipette after the contact), (**a4**) press Part B of the membrane against the curved tip using a transparency-covered clip to form a PR line on this tip, and (**a5**) fabricate another PR line on the other side of the micropipette using the same process, etch the Au film using the two PR lines as masking patterns, and generate two separate Au lines on the micropipette after the removal of the two PR lines. The second approach (cross-sectional views): (**b1**) Spin-coat a layer of PR on a membrane, (**b2**) place the PR-coated membrane over the micropipette and blow nitrogen to press this membrane against the micropipette (a PR line is formed on the micropipette after the separation of the membrane and the micropipette), and (**b3**) fabricate another PR line on the other side of the micropipette using the same process. Etch the Au film using the two PR lines as masking patterns, and generate two separate Au lines on the micropipette after the etching of the two PR lines [20]. Adapted from [20]. Permission obtained.

To overcome this constraint, our group utilized flexible polymeric films as photoresist (PR) and pattern carriers. In this approach, uniform contact pressure fields are delivered via mechanical clips or gas fluxes [19,20,70]. The underlying configuration of this technical lineage proceeds as follows: a localized layer of PR wires or target metallic features is pre-formed across a high-compliance carrier skin, such as a supple, single-sided magnetic tape polymer film. Subsequently, this carrier membrane is driven to undergo controlled elastic deformation against the outer boundaries of the micropipette. Interfacial adhesion differentials then force the photoresist lines to transfer in situ onto the varying conic walls as a conformal etching mask. As a result, this approach secures high-fidelity pattern construction across non-developable topologies (Figure 16; [20]).

### 6.2. Mechanical Clip-Assisted Conformal Transfer Processes

The first conformal transfer methodology developed by our group is the mechanical clip-assisted line transfer process [20]. To execute this, a standard glass micropipette is uniformly coated with a 100 nm Au film via thermal evaporation, while a localized PR microline is pre-formed onto a supple, single-sided commercial magnetic tape carrier. The primary transfer is achieved by aligning the PR-loaded tape over the pipette and deploying a custom mechanical clip equipped with a low-modulus elastomeric buffer padding under a protective transparency film. Translating smoothly from the uniform cylindrical tube toward the shrinking conic apex, the elastic buffer padding dynamically dampens localized displacement variations. This compliance ensures a zero-gap conformal contact against the gold-coated outer wall across the non-developable tapered zone. The interfacial adhesion between the target substrate layer and the PR film is significantly higher than that between the PR film and the magnetic tape backing. Driven by this adhesive contrast, gently lifting the carrier cleanly releases the continuous PR line onto the micropipette surface. The transferred pattern extends uniformly from the straight tube section all the way to the curved tip vertex (Figure 16(a1–a5); [20]).

### 6.3. Gas Flux-Assisted Conformal Transfer Processes

While clip-assisted transfer exhibits high reliability across standard topographies, it encounters a mechanical failure bottleneck when scaled to brittle, ultra-fine apexes. This limitation becomes particularly critical in single-cell electrophysiology, where glass tips feature outer diameters narrowing down to 1–5 μm. Even with elastomeric cushions, physical sliding motions introduce localized mechanical shear friction and concentrated non-uniform loads, frequently triggering structural snapping or micro-fractures along these fragile boundaries. To eradicate this hazard, an alternative contact-free approach was developed: the gas flux-assisted non-destructive transfer process [19,20]. Within this architecture, a contact-free, zero-mass, high-purity nitrogen gas flux is introduced to replace physical sliding clips as the transmission medium of compliant force.

In this protocol, the metallized glass micropipette and the flexible carrier skin pre-patterned with PR microlines are aligned in proximity. A digital gas-actuated delivery system equipped with a micro-scale nozzle is oriented over the assembly, directing a vertical, pressure-regulated jet. Driven along the axial track of the micropipette via a high-precision 3D scanning stage, the moving stream generates a gentle, uniformly distributed pressure field that deforms the supple carrier downward. Owing to the high spatial conformality of the gas stream, the flow field adaptively accommodates variable cross-sections as the outer diameter narrows from hundreds of micrometers down to the sub-micron scale. This establishes a zero-gap conformal contact across the ultra-fine tip without imposing localized bending moments or frictional wear (Figure 16(b1–b3)). Empirical characterizations demonstrated that this protocol successfully transferred unbroken, continuous “PR” lines onto the highly fragile outer walls of micropipettes with apex diameters measuring 5 μm, 2 μm, and down to a physical boundary of 1 μm without inducing any structural damage [19,20].

Following the compliance-driven transfer, the continuous PR wires on the 3D irregular glass outer walls serve as high-fidelity chemical masks. The holistic device is immersed within an iodine/potassium iodide liquid formulation for selective wet etching to strip the unprotected gold film, followed by an acetone bath to remove the remaining polymer. This process successfully yields continuous, sharp-edged, and directly functionalized Au conductive microlines in situ across the non-developable glass topologies [20].

### 6.4. Capillary Direct-Writing

Tracking the manufacturing evolution across the two-decade (2006–2026) timeline, the fundamental logic of sidewall patterning has experienced a large shift. Specifically, leveraging interfacial fluidic and aerodynamic compliance bypasses the geometric constraints of non-developable boundaries, enabling functional trace deposition along irregular vertical walls. This physics-driven strategy has undergone a digital, maskless transformation, crystallizing into modern Microfluidic Conformal Coating and Capillary Direct-Writing technologies [71,72,73]. 3D microfluidic coating builds upon classical concepts of boundary stress regulation while eliminating the need for solid elastomeric carrier skins. Rather than using pre-patterned solid stencils, current approaches deploy low-viscosity, high-surface-tension liquid functional inks. Advanced media like silver nanoparticle suspensions or liquid metal alloys are now applied through adaptive fluidic boundaries directly across non-developable interfaces [74,75,76].

Researchers implement a microfluidic capillary printing head capable of multi-axis synchronous 3D motion, guiding it to scan along 3D sidewalls featuring complex conical tapers, substantial slopes, or recessed steps. By delivering the liquid ink via computer-controlled microfluidic pumps, a dynamically stable capillary meniscus liquid bridge is formed between the scanning nozzle apex and the non-developable curved boundary [72,74]. As the printing head dynamically tracks the intricate spatial curvature of the irregular surface, fluidic forces regulate the contact interface in real time. Within the liquid meniscus bridge, Marangoni effects drive intense internal capillary suction and localized surface tension gradients. Functionally, these coordinated interfacial phenomena serve as a ‘soft digital fluidic clip’ to pin and guide the flowing ink [73,76].

This fluidic configuration self-regulates the ink’s wetting and pinning boundaries along steep vertical slopes. By suppressing gravitational sagging and fluidic collapse, it enables the *in situ* deposition of fine functional wiring traces directly onto non-developable planes and fragile tips [72,74]. Moreover, this maskless, continuous process induces negligible mechanical stress on delicate target architectures. By substantially expanding the spatial degrees of freedom for pattern replication, this fluidic compliance mechanism facilitates the development of multi-channel functional surgical instruments and non-planar wearable microneedle bio-chips [71,75,76].

## 7. Laser-Assisted Maskless and Local Direct Transfer

### 7.1. Tilted Interference Lithography

Section 3, Section 4, Section 5 and Section 6 reveal a unified physical foundation among the diverse technical lineages. Whether employing elastomeric viscoelastic drawing (Section 3), thermo-elastic strain recovery (Section 4), capillary-driven stencil wrapping (Section 5), or compliance-mediated pattern transfer (Section 6), these classical routes consistently fall within the domain of contact-based manufacturing. Throughout these frameworks, physical molds, polymer masks, sliding clips, or fluidic bridges must forge direct contact against vertical sidewalls or non-developable geometries to deliver localized mechanical loading or spatial blocking.

However, when confronted with the vertical boundaries of state-of-the-art semiconductor integrated chips, high-aspect-ratio MEMSs, and specialized aerospace micro-sensor components, contact-based methods reveal substantial deficits. These ultra-precision setups frequently feature highly fragile, released, or floating micro-scale cantilevers, membrane capacitors, or highly sensitive bio-active layers. Any subtle physical compressive stamping or sliding friction risks introducing irreversible mechanical stress fractures, surface micro-scratches, or catastrophic structural collapse. Concurrently, cyclic contact and demolding of elastomeric stamps inherently transfer organic polymer residue contamination and localized particle defects onto clean sidewall facets, which is fatal for electronics demanding zero-contamination manufacturing. To thoroughly cross this physical limit of contact and eliminate the spatial constraints of physical stencils, both scientific and industrial sectors have spearheaded a fundamental paradigm shift. Over the past two decades (2006–2026), researchers have advanced a completely contact-free, digital, and maskless direct transfer processing lineage. Driven by targeted photon energy beams, this strategy enables localized energy delivery and spatial direct writing across non-planar architectures.

**Figure 17:**
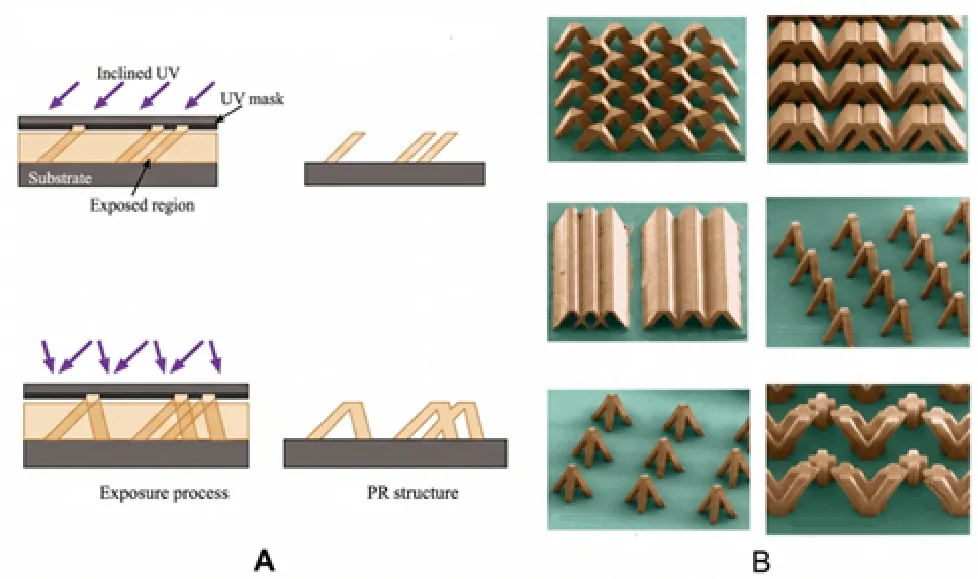
(**A**) Schematic diagram of oblique microstructure fabrication with single inclined/reflected UV exposure. (**B**) The positive oblique microstructures fabricated with several inclined UV exposures (adapted from [78]). Adapted from [78]. Permission obtained.

In the early phases of contact-free direct writing, researchers initially attempted to alter conventional optical pathways to bypass the exposure dead zones of vertical boundaries, developing tilted interference and holographic photolithography [77,78,79,80]. This framework implements high-precision multi-axis rotating stages, tiltable light sources, or specialized reflective micro-mirrors to modify the trajectory of collimated light beams. By dynamically shaping the optical path, light beams are directed into deep micro-trenches at precisely computed oblique angles θ (Figure 17A; [78,81]). This geometric control ensures uniform photon delivery onto steep, non-planar sidewalls. By overlapping multiple tilted rays in space, 3D holographic interference patterns are created, organizing periodic exposure intensity fields along the vertical sidewalls to execute *in situ* photochemical development without physical tool contact (Figure 17B; [79]). Nevertheless, standard optical radiation remains constrained by the fundamental optical diffraction limit. Additionally, due to narrow depth-of-focus windows, oblique single-photon beams undergo focus splitting or defocus blurring when traversing sharp 3D edge topologies. These intrinsic physical boundaries drove this technical lineage to evolve toward non-linear multi-photon processing. It has successfully branched into non-linear sub-diffraction regimes and microscopic kinetic droplet ejections [77].

Recent developments in 3D non-planar manufacturing have expanded microfluidic applications far beyond passive, static superhydrophobic drag reduction, transitioning toward active 3D liquid routing, continuous-flow manipulation, and field-programmable open-channel interfaces. By leveraging localized capillary pressure asymmetry, multi-curvature topographical gradients, or active stimulus-responsive coatings, non-planar surfaces enable multimodal liquid steering.

For instance, asymmetric fang-structured micro-architectures featuring bottom-up multi-curvature topologies induce directional Laplace pressure gradients, enabling *in situ*, multimodal liquid manipulation and autonomous multi-path fluidic routing based on surface tension variations [82]. To transition from static surface structures to dynamically adjustable microfluidics, reconfigurable hierarchical rectifiers integrated with responsive soft-magnetic micro-ratchets have been developed [83,84]. Under programmable static magnetic fields, these structures switch their morphology to achieve selective directional transport and biomimetic inchworm-like liquid crawling across three orders of magnitude in fluid volume [83,84].

Integrating thermoresponsive macromolecules (such as poly(N-isopropylacrylamide)) onto non-planar microstructures introduces dynamic wettability switching [85]. Interplay between local thermal gradients and structural heterogeneities surpasses traditional passive surface guidance. This active coupling drives complex fluidic phenomena, such as anti-gravity liquid climbing and real-time dynamic liquid patterning. It provides robust spatiotemporal manipulation over localized cascade chemical reactions [85]. Complementing open-surface spreading, 3D open fluidic networks fabricated via stereolithography utilize capillary-driven open polyhedral frames to route continuous flows in 3D space [86]. These open architectures eliminate rigid solid walls while retaining precise flow direction and speed control, significantly enhancing interfacial mass/heat transfer, programmable dual-modal sensing, and multi-step reaction synthesis [86].

### 7.2. Plasma-Assisted, Advanced Laser, and Photonic–Plasma Hybrid Sidewall Processing

Soft-membrane transfer and non-contact micro-jetting routes provide high material versatility across broad geometries. However, achieving sub-micron spatial features across steep inorganic topographies—such as single-crystalline Si, quartz, and refractory metals—presents distinct material challenges. Addressing these rigid surfaces requires the localized modulation of directional energetic particles and focused photon beams. To outline the technological domain, this section evaluates plasma-assisted processing, advanced laser-matter interactions, and emerging hybrid photonic–plasma manufacturing strategies tailored for non-planar geometries.

#### 7.2.1. Plasma-Based Sidewall Modification and Passivation Regimes

Plasma-based manufacturing utilizes reactive ion fluxes and high-density gaseous species to execute subtractive etching, additive plasma-enhanced chemical vapor deposition (PECVD), or functional surface chemistry modification along vertical facets. In directional reactive-ion etching and the classical Bosch process, alternating cycles of anisotropic fluorine-based etching (${\mathrm{SF}}_{6}$) and fluorocarbon passivation ($C_{4}F_{8}$) generate deep Si through-vias and high-aspect-ratio vertical channels [6]. During the passivation phase, a conformal hydrophobic polymer layer is deposited continuously onto 90° vertical sidewalls, protecting them from physical ion bombardment while subsequent directional ion fluxes sputter only the horizontal trench floors [6].

Beyond cyclic etching, plasma-assisted conformal functionalization allows uniform chemical activation across deep non-planar topographies. By modulating the gas pressure, ion sheath thickness, and substrate inclination angles relative to the electric field vector, plasma-induced polymerization can deposit nanometer-thin cross-linked polymer barriers along non-developable walls [87]. However, plasma-based sidewall processing is inherently constrained by local ion shadowing, charge-buildup-induced micro-trenching, and localized aspect-ratio-dependent etching lag, which alters the local etch rates across varying cavity depths [6].

#### 7.2.2. Advanced Laser-Based Regimes and Multi-Axis Processing

Laser-based processing operates through localized, contactless energy delivery, spanning subtractive ablation, surface nanostructuring, and additive material deposition. Based on the pulse duration, laser fluence, and material absorption cross-sections, laser sidewall processing resolves into distinct physical regimes:

When focused ultra-short picosecond or femtosecond pulses strike vertical or inclined boundaries at fluences exceeding the optical damage threshold, localized phase explosion and plasma ejection remove material with minimal heat-affected zones [88]. At sub-ablation fluences, optical interference between the incident laser field and surface-scattered electromagnetic waves triggers self-organized periodic structures, creating nanoscale ripples along non-planar facets for controlled cell guidance and altered surface wettability [88].

Focused ultraviolet or infrared laser lines drive the localized photo-reduction of metal salt precursors. Alternatively, this targeted beam activates pre-coated organometallic films to form continuous conductive features directly along vertical Si walls [89]. Following electroless plating, conductive metal lines form exclusively along the laser-scanned tracks without physical mask contact [89].

To overcome line-of-sight constraints across complex 3D topographies, advanced laser platforms incorporate multi-axis galvo-scanners, tiltable beam-steering optics, and high-precision 5-axis CNC positioning stages [90]. By maintaining the focal spot perpendicular to local surface tangents, these setups mitigate focal depth blurring and beam ellipticity distortions across curved boundaries [90].

#### 7.2.3. Photonic–Plasma Hybrid Processing Strategies

To bypass the standalone physical constraints of photon optical diffraction and plasma ion anisotropy, hybrid photonic–plasma processes integrate localized laser energy delivery with gaseous reactive-plasma environments. In laser-assisted plasma etching, a focused laser beam selectively heats or photo-excites specific micro-zones on a substrate immersed in a low-pressure reactive plasma. This localized thermal elevation accelerates the reaction kinetics between radical species and the target matrix, enabling maskless, spatially addressable deep etching along vertical boundaries at rates exceeding unassisted plasma processing.

Conversely, laser-assisted surface activation coupled with subsequent plasma-enhanced functionalization allows sequential multi-material integration [89]. The focused laser beam first pattern-activates or modifies local chemical bonding along a vertical wall, after which an isotropic plasma process selectively deposits functional organic or metallic layers exclusively on the laser-modified zones [88,89]. Hybrid routes successfully eliminate physical mask limits and improve spatial resolution across non-planar topographies. However, their industrial implementation introduces substantial systems-engineering challenges. Deploying these methods requires complex dual-source chamber integration, real-time multi-vector alignment, and careful mitigation of thermal stress buildup across heterogeneous material interfaces [90].

## 8. Analytical Comparison and Future Perspectives

### 8.1. Comprehensive Cross-Route Manufacturing Matrix Analysis

To provide a lucid, mechanism-based classification taxonomy, this section constructs a systematic manufacturing matrix (Table 2). Grounded in distinct mechanical and physical pattern transfer configurations, this evaluation matrix executes a multi-dimensional quantitative and qualitative cross-comparison. Specifically, it systematically evaluates the five core technical lineages reviewed across previous sections: mechanical elastomeric hot embossing, shape-memory self-driven contraction, flexible membrane capillary shadow masking, compliance-driven contact transfer, and contact-free digital photonic direct writing. Through this comparative synthesis, the fundamental trade-offs between processing throughput, spatial resolution, and geometric compatibility are distinctly illuminated. The evaluative metrics encompass spatial feature resolution limits, substrate material versatility, 3D topological constraint adaptability, manufacturing volumetric throughput, and critical 3D strain distortion rates.

From the perspective of solid mechanics and fluidic rheology boundary conditions, each individual fabrication lineage exhibits distinct trade-off characteristics:

1. Mechanical Deformation and Hot Embossing (Section 3): This route possesses high industrial processing throughput and low equipment capital, making it uniquely suited for fabricating large-scale, multi-surface microfluidic topographies. Because polymer deformation operates through macroscale visco-plastic displacement above thermal switching limits, shape stability is challenged. The combined action of tensile drawing and shear flow stretches the soft material along vertical interfaces. This non-uniform displacement causes non-negligible pattern elongation distortions across 3D sidewalls.
2. Shape-Memory Polymer Self-Driven Contraction (Section 4): This approach decouples the patterning cycle from the physical constraints of rigid tooling. It harvests internal material lattice strain-recovery stresses to self-drive feature relocation under zero external force fields, presenting immense mechanical value for safeguarding fragile 3D components. Nonetheless, this route remains substantially restricted by substrate material limitations, requiring specialized pre-stretched polymers. Furthermore, the high-ratio volumetric shrinkage inherent to this process generates intense mechanical stress during contraction. Driven by elastic mismatch instabilities across heterogeneous boundaries, this localized stress frequently introduces microscopic interfacial wrinkling patterns.
3. Flexible Membrane-Mediated Shadow Stenciling (Section 5): This strategy successfully breaks processing blind spots across inorganic rigid semiconductor sidewalls. This route utilizes capillary surface tension to drive an ultra-thin through-hole film to conformally self-wrap along vertical crystalline Si facets. Guided by a precision 3D vector geometric projection model, this physical interaction secures high-fidelity microscale patterning over non-deformable matrices. However, its primary barrier stems from the delicate manual tensioning and alignment manipulation required for ultra-thin stencils, substantially restricting its adaptation for multi-tier nested overlay routing.
4. Compliance-Driven Membrane Transfer (Section 6): This protocol was specifically engineered to resolve non-developable architectures, such as the fragile, variable-cross-section conic apexes of glass micropipettes. Implementing compliant sliding clips or contact-free aerodynamic digital nitrogen gas fluxes as soft force-delivery vectors overcomes concentrated bending moments and shear friction failures. Its structural constraint lies in the prerequisites of pre-patterning a 2D polymer carrier skin, rendering it a localized routing protocol unsuitable for wafer-scale, high-density texturing.
5. Contact-Free Laser-Assisted Direct Transfer (Section 7): This technical lineage represents advanced spatial degrees of freedom in modern 3D manufacturing. These photon-driven pathways eliminate physical mask boundaries and mechanical tool collisions. Their primary costs scale into substantial laser system instrumentation expenditures and constrained volumetric throughput tied to point-by-point or droplet-by-droplet digital scanning modes.

### 8.2. Manufacturability and Technology Readiness Level Assessment

Non-planar and sidewall patterning techniques frequently demonstrate high spatial resolution and design flexibility in academic settings. However, translating these laboratory methodologies into industrial-scale manufacturing introduces distinct scalability hurdles. Achieving commercial viability mandates a rigorous evaluation of throughput, yield, defect density, and equipment compatibility. To provide a pragmatic roadmap for process selection, the fabrication routes reviewed in this work are evaluated across five distinct Technology Readiness Levels (TRLs) (See Table 3):

1. Conceptual Feasibility and Laboratory Proof of Concept (TRL 2–3)

Emerging additive direct-write routes, such as multi-axis laser-induced forward transfer, field-driven electrohydrodynamic printing, and active stimulus-responsive printing, currently operate primarily at TRL 2–3. These techniques demonstrate fundamental physical feasibility and intricate 3D pattern placement on local micro-features. However, they are inherently constrained by serial point-by-point processing speeds (typically $10^{-4}–10^{-2}\;{\mathrm{cm}}^{2}/\min$), high susceptibility to localized dynamic instability, and limited batch-to-batch repeatability.

2. Repeated Device Fabrication and Small-Batch Demonstration (TRL 3–4)

Techniques utilizing shape-memory polymer substrates, compliant elastomeric balloon stamps, and flexible shadow-mask stenciling have advanced to TRL 3–4. These manufacturing methods enable the reproducible fabrication of high-performance functional prototype devices. Established demonstrations include conformal sensor arrays, curved imagers, and 3D microfluidic routing networks. These architectures can be reliably patterned across active areas spanning from several square millimeters to tens of square centimeters. Key process bottlenecks at this stage center on pattern placement errors induced by non-uniform viscoelastic deformation, interfacial delamination during stamp release, and thermal expansion mismatches during cycle loading.

3. Wafer-Scale and Pilot Manufacturing Potential (TRL 4–5):

Advanced non-planar routes—including mechanical micropunching, Si vertical serpentine interconnects with vacuum conformal transfer, and DRIE passivation—have achieved mature Technology Readiness Levels (TRL 4–5). These platforms leverage established semiconductor infrastructure, including automated bonders and dry etchers. This compatibility facilitates high manufacturing yields (exceeding 90% under foundry-standard baselines), wafer-scale throughput, and long-term durability against cyclic mechanical loading [91].

### 8.3. Fundamental Physical and Technical Constraints in Sidewall Patterning

Each standalone fabrication route has achieved substantial functional integration milestones along vertical boundaries through innovative structural designs. However, a holistic synthesis of the sidewall micropatterning domain reveals persistent engineering boundaries. Specifically, three primary technical bottlenecks currently constrain the transition from laboratory prototypes to large-scale industrial manufacturing.

#### 8.3.1. 3D Multi-Planar Corner Interconnect Reliability

Across all 3D wiring tracks, conductive interconnect lines must continuously traverse from flat top horizons, navigate across sharp rigid {111} step edges, and extend down onto vertical boundaries or trench floors. These discontinuous 3D multi-planar corner junctions act as the weakest link regarding overall electromechanical reliability.

- Mechanical and Strain-Driven Lineages: During mechanical embossing and strain-driven contractive relocation, the corner vertexes undergo immense stress concentrations, frequently introducing microscale tensile-shear tearing of the functional metal film.
- Contact-Free Photonic Configurations: Within contact-free photonic pathways, sharp protruding edges introduce substantial diffraction distortions or cause arriving liquid micro-droplets to experience chaotic impact scattering and rebounding. This fluid-dynamic instability leads to substantial localized film thinning effects or the nucleation of micro-cracks.

Under long-term cyclic electrical operations, coefficient of thermal expansion (CTE) mismatches generate substantial localized electro-thermal strains. These thermal–mechanical stresses readily trigger catastrophic electromigration blowouts or fatigue delamination precisely at multi-planar intersections. Consequently, these structural degradation modes induce total open-circuit failure across the 3D circuit layout.

#### 8.3.2. Strain-Induced Pattern Elongation Distortion and Metrology Control

Relocating or stretching a 2D planar feature onto a vertical 3D sidewall plane is fundamentally a highly non-linear finite-strain solid mechanics deformation process. This physical process accompanies the spatial migration of materials, encompassing thermoplastic substrates, flexible skins, and liquid-phase metallic streams. During transfer, micro-features that originally possess ideal geometric boundaries—such as square tracks or circular dots—undergo substantial structural alteration. As these features cross vertical wall faces, they suffer substantial tensile elongation, asymmetrical stretching, and spatial widening.

To offset these topographies, ultra-precision 3D strain-compensation algorithms must be formulated. These mathematical models pre-incorporate inverse geometric distortions directly into the initial 2D mask layout. By doing so, they effectively neutralize the tensile stretching forces encountered during subsequent rheological workflows, although achieving such algorithmic precision remains a highly challenging engineering task. Conventional industrial metrology platforms—such as collimated split-field microscopes and automated optical alignment systems—have their fields of view and focal planes confined to horizontal planes. Consequently, when patterning features across varying depths along a vertical wall, these systems encounter substantial depth-of-focus limitations. This limitation disables automated, large-scale, *in situ*, inline precision overlay error measurements, substantially restricting high-precision multi-layer overlay processing of functional sidewall architectures.

#### 8.3.3. Long-Term Interfacial Stability of Heterogeneous Materials

Sidewall manufacturing inherently demands the tight integration of multi-class, heterogeneous functional materials. It forces rigid noble metals (Au, Ag), single-crystalline Si, amorphous polymers, and responsive hydrogels onto a shared platform. Managing these contrasting material classes within highly confined vertical spaces poses a fundamental interfacial challenge. Structural shaping leaves vertical sidewalls burdened with high residual shear stresses. These localized stresses degrade interfacial energetics along non-planar boundaries. As a result, the atomic-level orbital overlap and chemical bonding strength across these heterogeneous junctions fall well below the levels achievable on flat horizontal substrates.

Under harsh operational settings—marked by humidity, alternating electric fields—fluidic shearing, and thermal fluctuations, these non-planar heterogeneous interfaces undergo substantial physical degradation. Specifically, these environmental stressors induce localized interfacial stress relaxation, microscopic moisture seepage, and chemical stress-corrosion delamination. These degradation modes trigger structural collapse or parameter drift, leaving the long-term operational lifespans of these 3D systems unable to match those of mature wafer-scale 2D planar integrated circuits.

### 8.4. Future Horizons: Convergence Towards Multi-Dimensional, Multi-Material Hybrid Manufacturing Systems

Reflecting on the technical evolution of 2000–2025, non-planar 3D micro/nanofabrication has completed its initial development phase of proving baseline manufacturing feasibility [92]. Looking ahead to next-generation manufacturing blueprints, standard standalone methodologies will no longer suffice. Addressing persistent physical bottlenecks and strategic application demands necessitates broader technological convergence. Moving forward, this domain must march toward total integration into multi-dimensional, multi-material hybrid manufacturing systems. To bridge present demonstrations with high-impact applications, concrete technological roadmaps are established across three primary domains:

#### 8.4.1. Technological Roadmap for 3D Quantum Computing Architectures

To realize scalable 3D quantum processors, non-planar patterning must address high-aspect-ratio Through-Silicon Vias (TSVs) and 3D superconducting interconnects [93]. The technical roadmap requires the following:

(I) Conformal Dielectric Passivation: Deposition of ultra-uniform, low-loss dielectric layers (e.g., ${\mathrm{Al}}_{2}O_{3}$, ${\mathrm{SiN}}_{x}$ via ALD/PECVD) along vertical TSV sidewalls with aspect ratios exceeding 20:1 to minimize dielectric loss tangents.

(II) Superconducting Trace Patterning: This frontier application relies on direct-write jetting or compliance-driven transfer of high-performance superconducting metal films (e.g., Al, Nb, or TiN). These specialized materials are conformally patterned onto 3D vertical facets and non-planar interconnect bridges. By maintaining sub-μm edge resolution, this approach delivers the precise spatial control needed to support high-fidelity flux-qubit coupling and 3D microwave routing [94].

#### 8.4.2. Technological Roadmap for Bio-Adaptive Smart In Vivo Micro-Probes

Deploying neural and physiological probes onto high-curvature biological interfaces demands biomechanically compatible, non-coplanar manufacturing [95,96]:

(I) Conformal Flexible Encapsulation: This involves utilizing pinhole-free, bio-inert polymeric coatings (e.g., Parylene-C, SU-8) as insulating envelopes over 3D needle surfaces and micro-pipette contours.

(II) Apex Multi-Channel Electrode Routing: This biomedical route achieves non-coplanar, multi-site micro-electrode array patterning directly onto sub-10μm needle apexes and flexible shafts. By integrating conductive pathways with micro-channels, it enables simultaneous localized electrophysiological sensing and drug-delivery microfluidics. These non-planar interfaces possess good mechanical adhesion, preventing delamination during deep tissue insertion [96].

#### 8.4.3. Technological Roadmap for Autonomous Multi-Functional Micro-Robotics

For untethered 3D micro-swimmers and bio-hybrid micro-actuators, hybrid manufacturing must unify structural morphing and functional excitation [97]:

(I) Magnetic Domain Patterning: Applying direct-writing magnetic nanoparticle inks (e.g., $\mathrm{NdFeB}/{\mathrm{Fe}}_{3}O_{4}$ suspended in elastomers) onto non-planar elastomeric frames, followed by localized magnetic field alignment to program multi-axis magnetic domain orientation [83].

(II) Integrated Micro-Actuator Hetero-Integration: Combining shape-memory polymers, piezoelectric thin films, and conductive liquid-metal tracks onto 3D open polyhedral frames, establishing autonomous propulsion and environmental sensing in complex biological fluids.

## 9. Conclusions

Sidewall patterning has emerged as an important enabling technology for extending micro/nanofabrication from conventional planar surfaces to 3D architectures. By exploiting previously underutilized vertical and non-planar surfaces, sidewall patterning substantially increases the available functional area for electronic, photonic, microfluidic, sensing, and bio-integrated systems, thereby opening new opportunities for 3D integration and multifunctional device design.

This review has classified existing sidewall-patterning techniques into two fundamental categories according to the pattern-generation mechanism: pattern relocation and direct physical/chemical modification. Pattern-relocation methods, including hot embossing, shape-memory-assisted transfer, compliant membrane transfer, and micro-transfer printing, relocate pre-fabricated patterns onto non-planar surfaces while largely preserving the performance of conventional planar fabrication. In contrast, direct physical/chemical modification methods, such as flexible shadow masking, laser-assisted direct writing, and laser-induced forward transfer, generate patterns directly on sidewall surfaces and offer greater geometric freedom for complex 3D structures. Each category exhibits distinct strengths and limitations in terms of spatial resolution, substrate compatibility, geometric adaptability, manufacturing throughput, and process complexity, and no single technique simultaneously satisfies all performance requirements.

Although remarkable progress has been achieved during the past two decades, several fundamental challenges remain. Reliable electrical interconnection across sharp 3D corners, deformation-induced pattern distortion during transfer, precise multilayer alignment on non-planar surfaces, and the long-term reliability of heterogeneous material interfaces continue to limit manufacturing yield and large-scale implementation. In addition, balancing high-resolution patterning with wafer-scale throughput remains a major obstacle to industrial adoption. Addressing these issues will require advances in mechanics-guided process design, 3D metrology, materials engineering, and manufacturing automation.

Future developments are expected to move beyond individual fabrication techniques toward integrated hybrid manufacturing platforms that combine the complementary advantages of multiple approaches. The convergence of transfer printing, direct-write manufacturing, advanced lithography, additive manufacturing, and intelligent process control will enable increasingly complex 3D heterogeneous systems with improved accuracy, scalability, and reliability. Such hybrid strategies are expected to play an essential role in next-generation applications including advanced semiconductor packaging, 3D integrated circuits, quantum information hardware, flexible and wearable electronics, implantable biomedical devices, intelligent microfluidic systems, and autonomous microrobotics.

Overall, sidewall patterning has evolved from a niche fabrication capability into a fundamental manufacturing paradigm for 3D micro/nanosystems. Continued advances in fabrication science, materials, and process integration are expected to transform sidewall patterning into a mature manufacturing technology, enabling highly integrated multifunctional microsystems that effectively exploit the 3D design space beyond conventional planar fabrication.

## Figures and Tables

**Table 1:** Inherent limitations of classical lithographic and planar non-lithographic technologies across 3D non-planar surfaces.

Lithography Class	Specific Method	Pattern Transfer Mechanism	Failure Mechanism for Sidewalls	Key References
Photolithographic	Standard UV & E-Beam Lithography	Unidirectional collimated energy ray exposure	The incident beam runs parallel to vertical facets, yielding near-zero photon flux interception (θ≈0°), while finite depth-of-field (DOF) constraints induce focal blurring across stepped topographies.	[22,25]
Non- Photolithographic	Scanning Probe Lithography (e.g., DPN)	Cantilever probe tip translation across planar coordinates	Kinematic constraints; physical collisions between the cantilever assembly and upper step edges prevent tip engagement along steep vertical facets.	[26,29]
Non- Photolithographic	Standard Soft Lithography	Compliant elastomeric stamp pressing along the normal axis	Geometric trapping during normal engagement; localized stress concentration at sharp boundaries induces stamp buckling and roof collapse, preventing conformal contact.	[27,32]
Non- Photolithographic	Nanoimprint Lithography (NIL)	Rigid mold compression of thermal/UV-softened polymers	Unidirectional loading yields zero normal stress on vertical facets ($\sigma_{n}=0$), failing to generate the lateral shear pressure ($\tau_{xy}$) required for horizontal mass transport.	[28,33]

**Table 2:** Comparison of Sidewall Patterning Methods.

Technical Category	Specific Method & Primary References	Min. Feature Size	Substrate/Trench Scale	Material Compatibility	Quantified Throughput & Scalability	Primary Process Limitations & Distortion Mechanisms
Section 3: Elastomeric Hot Embossing	Micropunching Lithography, Si-reinforced masters [9]	10 μm	Aspect ratio up to ∼0.3:1; 100 μm step height	Thermoplastics (PMMA, PC, PS), polymeric blends	Wafer-level parallel embossing (>10 cm^2^ area)	Tool mechanical deflection, thermal expansion mismatch, viscoelastic elongation distortion.
Section 4: Shape Memory Polymers	Pre-stretched PS block dual-recovery transfer [17]	10 μm	In-plane shrinkage ∼95%; thickness up to 40×	Pre-stretched PS, conductive metallic tracks	Batch thermal-cycle process (minutes/cycle)	Stress concentration at trench corners; Young’s modulus mismatch induces micro-wrinkling.
Section 5: Stenciling/Shadow Masking	Hexane-assisted through-hole films, tilted vapor [19]	10 μm	Conformal over 110 μm vertical Si sidewalls	Crystalline Si, rigid inorganic substrates, evaporated metals	Wafer-level parallel PVD evaporation	Line-of-sight vapor trajectory tilt causes edge blurring and shadow distortion.
Section 6: Compliance-Driven Transfer	Clip-assisted sliding, gas-flux-assisted, printing [19,20], and capillary writing [71,72,73]	5 μm	Curved conic tips down to 5 μm outer diameter	Fragile glass cones, ceramics, elastomers, liquid metals	Not reported (fixture-assisted single-chip process)	Mechanical clips introduce localized bending moments, risking fragile substrate fracture.
Section 7.1: Tilted Interference Lithography	Tilted exposure [77,78,79]	10 μm	3D overhangs, deep cavities, 50–150 μm air gaps	Photopolymers, metal nano-inks, liquid metals, hydrogels	Serial point-by-point process (<1 $\;{\mathrm{mm}}^{2}/\min$)	Low throughput bottleneck of serial point-by-point writing, localized heat-affected zone.
Section 7.2: Plasma-Based Processing	DRIE Bosch process & low-temp poly-SiGe micromachining [6]	50 nm–10 μm	Deep Si through-vias, 90° vertical channels (>20:1)	Single-crystal Si, quartz, poly-SiGe, inorganic matrices	Wafer-level batch processing (high throughput)	Aspect-ratio-dependent etching lag, local ion shadowing, dielectric charge buildup.
Section 7.2: Photonic–Plasma Hybrid	Laser-assisted plasma etching and localized activation [89]	100 nm–5 μm	Deep vertical cavities and non-planar 3D boundaries	Semiconductors, ceramics, multi-material heterostructures	Not reported (experimental laboratory setups)	Complex dual-source chamber integration, dual-vector alignment challenges, thermal stress.

**Table 3:** Process Manufacturability and Technology Readiness Level Classification.

Fabrication Route	Primary Manufacturing Bottleneck	Area Throughput & Scalability Category	Process Yield & Repeatability Range	Estimated TRL ^a^	Target Development Stage
Mechanical Micropunching & Embossing	Tool mechanical wear; demolding-induced viscoelastic shear stress	High (wafer-level parallel/batch)	Not reported (lab proof of concept)	TRL 3–4	Laboratory Prototype Validation
Shape-Memory Polymer Origami	Thermal recovery kinetics; maximum elastic strain boundaries	Low to Moderate (thermal-cycle/ batch)	Not reported (lab proof of concept)	TRL 2–3	Academic Proof of Concept
Compliant/Balloon Transfer Printing	Viscoelastic stamp deformation; localized contact placement error	Moderate (area-selective batch)	Not reported (lab proof of concept)	TRL 3–4	Specialized Niche Fabrication
Flexible Shadow Masking/Stenciling	Mask aperture clogging; vapor line-of-sight trajectory blurring	Moderate to High (batch PVD vapor)	Not reported (lab proof of concept)	TRL 3–4	Specialized Laboratory Device
Direct Laser Writing	Serial point-by-point exposure speed; heat-affected zone	Low (serial point-by-point scanning)	Not reported (high localized fidelity)	TRL 2–3	Custom Prototype Direct-Write
Si Vertical Interconnects & Vacuum Conformal Transfer	Multi-layer lithographic alignment complexity	High (wafer-level batch execution)	Moderate (foundry-compatible integration)	TRL 4–5	Laboratory Prototype

^a^ TRL Definitions: TRL 2 ≈ technology concept formulated; TRL 3 ≈ experimental proof of concept; TRL 4 ≈ technology validated in lab environment; TRL 5 ≈ technology validated in relevant environment.

## Data Availability

No new data were created or analyzed in this study. Data sharing is not applicable to this article.

## References

[1] Esashi M. (2010). Wafer-level packaging for MEMS. J. Micromech. Microeng..

[2] Knickerbocker J.U., Andry P.S., Dang B., Horton R.R., Patel C.S., Tsang C.K., Webb B.C. (2008). Three-dimensional silicon integration. IBM J. Res. Dev..

[3] Garrou P., Garrou P., Bower C.A., Ramm P. (2008). Introduction to 3D integration. Handbook of 3D Integration: Technology and Applications of 3D Integrated Circuits.

[4] Rogers J.A., Someya T., Huang Y. (2010). Materials and mechanics for stretchable electronics. Science.

[5] Gates B.D., Xu Q., Stewart M., Ryan D., Willson C.G., Whitesides G.M. (2005). New approaches to nanofabrication: Molding, printing, and other techniques. Chem. Rev..

[6] Franke A.E., Heck J.M., King Liu T.J., Howe R.T. (2003). Polycrystalline silicon-germanium films for integrated microsystems. J. Microelectromech. Syst..

[7] Kim D.H., Lu N., Ma R., Kim Y.S., Kim R.H., Wang S., Wu J., Won S.M., Tao H., Islam A. (2011). Epidermal electronics. Science.

[8] Lau J. (2011). Overview and outlook of through-silicon via (TSV) and 3D integrations. Microelectron. Int..

[9] Liu X., Luo C. (2009). Fabrication of Au sidewall micropatterns using Si-reinforced PDMS molds. Sens. Actuators A Phys..

[10] Thelander C., Agarwal P., Brongersma S., Eymery J., Feiner L.F., Forchel A., Scheffler M., Riess W., Ohlsson B.J., Gösele U. (2006). Nanowire-based one-dimensional electronics. Mater. Today.

[11] Huang Y., Duan X., Wei Q., Lieber C.M. (2001). Directed assembly of one-dimensional nanostructures into functional networks. Science.

[12] Ismach A., Segev L., Wachtel E., Joselevich E. (2004). Atomic-step-templated formation of single wall carbon nanotube patterns. Angew. Chem. Int. Ed..

[13] Quéré D. (2005). Non-sticking drops. Rep. Prog. Phys..

[14] Rothstein J.P. (2010). Slip on superhydrophobic surfaces. Annu. Rev. Fluid Mech..

[15] Liu X., Luo C. (2010). Fabrication of super-hydrophobic channels. J. Micromech. Microeng..

[16] Luo C. (2010). Generation of micropatterns on side surfaces of polymer and Si substrates using a micropunching lithography approach. Lithography: Principles, Processes and Materials.

[17] Liu X., Chakraborty A., Luo C. (2010). Fabrication of micropatterns on the sidewalls of a thermal shape memory polystyrene block. J. Micromech. Microeng..

[18] Wang H., Chakraborty A., Luo C. (2010). Fabrication of Au micropatterns on vertical Si sidewalls using flexible PDMS shadow masks. J. Micromech. Microeng..

[19] Wang H., Luo C. (2011). Generation of Au micropatterns on two sidewalls of a Si channel through a PDMS shadow mask. J. Micromech. Microeng..

[20] Wang H., Shen Y., Luo C. (2011). Two simple approaches to fabricate Au microlines on the outer surfaces of micropipettes. Microsyst. Technol..

[21] Kagawa G., Takahashi H. (2024). Liquid-immersion inclined-rotated exposure system for fabricating three-dimensional microstructures with large inclination angles. J. Micromech. Microeng..

[22] Madou M.J. (2011). Fundamentals of Microfabrication and Nanotechnology: Manufacturing Techniques for Microfabrication and Nanotechnology.

[23] Hui C.Y., Jagota A., Lin Y.Y., Kramer E.J. (2002). Constraints on microcontact printing imposed by stamp deformation. Langmuir.

[24] Brunner T.A. (2003). Why optical lithography will live forever. J. Vac. Sci. Technol. B Microelectron. Nanometer Struct. Process. Meas. Phenom..

[25] Del Campo A., Greiner C. (2007). SU-8: A photoresist for high-aspect-ratio and 3D submicron lithography. J. Micromech. Microeng..

[26] Piner R.D., Zhu J., Xu F., Hong S., Mirkin C.A. (1999). “Dip-pen” nanolithography. Science.

[27] Xia Y., Whitesides G.M. (1998). Soft lithography. Angew. Chem. Int. Ed..

[28] Chou S.Y., Krauss P.R., Renstrom P.J. (1996). Imprint lithography with 25-nanometer resolution. Science.

[29] Garcia R., Knoll A.W., Riedo E. (2014). Advanced scanning probe lithography. Nat. Nanotechnol..

[30] Salaita K., Wang Y., Mirkin C.A. (2007). Applications of dip-pen nanolithography. Nat. Nanotechnol..

[31] Luo C., Chakraborty A. (2009). Effects of dimensions on the sensitivity of a conducting polymer microwire sensor. Microelectron. J..

[32] Delamarche E., Schmid H., Michel B., Biebuyck H. (1997). Stability of molded polydimethylsiloxane microstructures. Adv. Mater..

[33] Heyderman L.J., Schift H., David C., Gobrecht J., Schweizer T. (2000). Flow behaviour of thin polymer films used for hot embossing lithography. Microelectron. Eng..

[34] Unno N., Mäkelä T. (2023). Thermal nanoimprint lithography—A review of the process, mold fabrication, and material. Nanomaterials.

[35] Poddar R., Luo C. (2006). A novel approach to fabricate a PPy/n-type Si junction. Solid-State Electron..

[36] Liu X., Luo C. (2007). An intermediate-layer method for producing metal micropatterns. J. Vac. Sci. Technol. B Microelectron. Nanometer Struct. Process. Meas. Phenom..

[37] Luo C. (2009). Generation of micropatterns of conducting polymers and aluminum using an intermediate-layer lithography approach and some applications. Microsyst. Technol..

[38] Chakraborty A., Luo C. (2009). Multiple conducting polymer microwire sensors. Microsyst. Technol..

[39] Luo C., Poddar R., Liu X. (2006). An innovative intermediate-layer lithography approach to replicate micropatterns in a conducting polymer. J. Vac. Sci. Technol. B Microelectron. Nanometer Struct. Process. Meas. Phenom..

[40] Chakraborty A., Liu X., Luo C. (2012). Micropunching lithography for generating micro- and submicron-patterns on polymer substrates. J. Vis. Exp..

[41] Chakraborty A., Liu X., Parthasarathi G., Luo C. (2007). An intermediate-layer lithography method for generating multiple microstructures made of different conducting polymers. Microsyst. Technol..

[42] Liu X., Chakraborty A., Luo C., Martingale J.P. (2008). Generation of all-polymeric diodes and capacitors using an innovative intermediate-layer lithography. Progress in Solid State Electronics Research.

[43] Whitesides G.M., Ostuni E., Takayama S., Jiang X., Ingber D.E. (2001). Soft lithography in biology and biochemistry. Annu. Rev. Biomed. Eng..

[44] Choe D., Jeong S.H., Baig C. (2024). Structural, topological, and rheological characteristics of entangled short-chain branched polymer melts under shear flow in comparison with the linear analog. J. Rheol..

[45] Suh K.Y., Park M.C., Kim P. (2009). Capillary force lithography: A versatile tool for structured biomaterials interface towards cell and tissue engineering. Adv. Funct. Mater..

[46] Luo C., Meng F., Francis A. (2006). Fabrication and application of silicon-reinforced PDMS masters. Microelectron. J..

[47] Schmid H., Michel B. (2000). Siloxane polymers for high-resolution, high-accuracy soft lithography. Macromolecules.

[48] Di Carlo D. (2009). Inertial microfluidics. Lab Chip.

[49] Carlson A., Bowen A.M., Huang Y., Nuzzo R.G., Rogers J.A. (2012). Transfer printing techniques for materials assembly and micro/nanodevice fabrication. Adv. Mater..

[50] Rogers J.A., Lagally M.G., Nuzzo R.G. (2011). Synthesis, assembly and applications of semiconductor nanomembranes. Nature.

[51] Kim D.H., Viventi J., Amsden J.J., Xiao J., Vigeland L., Kim Y.S., Blanco J.A., Panilaitis B., Frechette E.S., Contreras D. (2010). Dissolvable films of silk fibroin for ultrathin conformal bio-integrated electronics. Nat. Mater..

[52] Meitl M.A., Zhu Z.T., Kumar V., Lee K.J., Feng X., Huang Y.Y., Suh I., Deardorff M.M., Nuzzo R.G., Rogers J.A. (2006). Transfer printing by kinetic control of adhesion to elastomeric stamps. Nat. Mater..

[53] Lendlein A., Kelch S. (2002). Shape-memory polymers. Angew. Chem. Int. Ed..

[54] Liu Y., Boyles J.K., Genzer J., Dickey M.D. (2012). Self-folding of polymer sheets using local light absorption. Soft Matter.

[55] Kim J., Lee M., Shim H.J., Ghaffari R., Cho H.R., Son D., Jung Y.H., Soh M., Choi C., Jung S. (2014). Stretchable silicon nanoribbon electronics for skin prosthesis. Nat. Commun..

[56] Xu S., Yan Z., Jang K.I., Huang W., Fu H., Kim J., Wei Z., Flavin M., McCracken J., Wang R. (2015). Assembly of micro/nanomaterials into complex, three-dimensional architectures by compressive buckling. Science.

[57] Fu C., Grimes A., Long M., Ferri C., Rich B., Ghosh S., Ghosh S., Lee L., Gopinathan A., Khine M. (2009). Tunable nanowrinkles on shape memory polymer sheets. Adv. Mater..

[58] Bowden N., Brittain S., Evans A.G., Hutchinson J.W., Whitesides G.M. (1998). Spontaneous formation of ordered structures in thin films of metals supported on an elastomeric polymer. Nature.

[59] Stafford C.M., Harrison C., Beers K.L., Karim A., Amis E.J., VanLandingham M.R., Kim H.C., Volksen W., Miller R.D., Simonyi E.E. (2004). A buckling-based metrology for measuring the elastic moduli of polymeric thin films. Nat. Mater..

[60] Khang D.Y., Jiang H., Huang Y., Rogers J.A. (2006). A stretchable form of single-crystal silicon for high-performance electronics on rubber substrates. Science.

[61] Randall C.L., Gultepe E., Gracias D.H. (2011). Self-folding devices and materials for biomedical applications. Trends Biotechnol..

[62] Leong T.G., Lester P.A., Koh T.L., Call E.K., Gracias D.H. (2007). Surface tension-driven self-folding polyhedra. Langmuir.

[63] Cho J.H., Keung M.D., Verellen N., Lagae L., Moshchalkov V.V., Van Dorpe P., Gracias D.H. (2011). Nanoscale origami for 3D optics. Small.

[64] Vazquez-Mena O., Sannomiya T., Villanueva L.G., Voros J., Brugger J. (2011). Metallic nanodot arrays by stencil lithography for plasmonic biosensing applications. ACS Nano.

[65] Jackman R.J., Duffy D.C., Cherniavskaya O., Whitesides G.M. (1999). Using elastomeric membranes as dry resists and for dry lift-off. Langmuir.

[66] Py C., Reverdy P., Doppler L., Bico J., Roman B., Baroud C.N. (2007). Capillary origami: Spontaneous wrapping of a droplet with an elastic sheet. Phys. Rev. Lett..

[67] Hawker N., Ventikos Y. (2012). Interaction of a strong shockwave with a gas bubble in a liquid medium: A numerical study. J. Fluid Mech..

[68] Hawkeye M.M., Brett M.J. (2007). Glancing angle deposition: Fabrication, properties, and applications of micro- and nanostructured thin films. J. Vac. Sci. Technol. A Vac. Surf. Film..

[69] Gao C., Xu Z.C., Deng S.R., Wan J., Chen Y., Liu R., Huq E., Qu X.P. (2011). Silicon nanowires by combined nanoimprint and angle deposition for gas sensing applications. Microelectron. Eng..

[70] Ko H.C., Stoykovich M.P., Song J., Malyarchuk V., Choi W.M., Yu C.J., Geddes J.B., Xiao J., Wang S., Huang Y. (2008). A hemispherical electronic eye camera based on compressible silicon optoelectronics. Nature.

[71] Cao J., Li X., Liu Y., Zhu G., Li R.W. (2023). Liquid metal-based electronics for on-skin healthcare. Biosensors.

[72] Seol S.K., Kim D., Lee S., Kim J.H., Chang W.S., Kim J.T. (2015). Electrodeposition-based 3D printing of metallic microarchitectures with controlled internal structures. Small.

[73] Zheng Y., He Z., Gao Y., Liu J. (2013). Direct desktop printed-circuits-on-paper flexible electronics. Sci. Rep..

[74] Pannier C.P., Wang Z., Hoelzle D.J., Barton K.L. Electrohydrodynamic jet printing: A 3D printing technique for sensor fabrication. Proceedings of the Solid-State Sensors, Actuators, and Microsystems Workshop (Hilton Head 2016).

[75] Rusling J.F. (2018). Developing microfluidic sensing devices using 3D printing. ACS Sens..

[76] Zhang Y., Duan H., Li G., Peng M., Ma X., Li M., Yan S. (2022). Construction of liquid metal-based soft microfluidic sensors via soft lithography. J. Nanobiotechnol..

[77] Gonzalez-Hernandez D., Varapnickas S., Bertoncini A., Liberale C., Malinauskas M. (2023). Micro-optics 3D printed via multi-photon laser lithography. Adv. Opt. Mater..

[78] Han M., Lee W., Lee S.K., Lee S.S. (2004). 3D microfabrication with inclined/rotated UV lithography. Sens. Actuators A Phys..

[79] Jang J.H., Ullal C.K., Maldovan M., Gorishnyy T., Kooi S., Koh C., Thomas E.L. (2007). 3D micro- and nanostructures via interference lithography. Adv. Funct. Mater..

[80] Campbell M., Sharp D.N., Harrison M.T., Denning R.G., Turberfield A.J. (2000). Fabrication of photonic crystals for the visible spectrum by holographic lithography. Nature.

[81] Shiba S.F., Wang K., Kim J.J. Tiltable UV-LED lithography for 3D microfabrication. Proceedings of the 2021 21st International Conference on Solid-State Sensors, Actuators and Microsystems (Transducers).

[82] Sun S., Zhang Y., Wu S., Wang L. (2024). In situ multi-directional liquid manipulation enabled by 3D asymmetric fang-structured surface. Adv. Mater..

[83] Kim Y., Yuk H., Zhao R., Chester S.A., Zhao X. (2018). Printing ferromagnetic domains for untethered fast-transforming soft materials. Nature.

[84] Miao J., Tsang A.C.H. (2024). Reconfigurability-encoded hierarchical rectifiers for versatile 3D liquid manipulation. Adv. Sci..

[85] Miao J., Li J., Tsang A.C.H. (2025). Competition and synergy in programmable open microfluidics: Thermal fields vs structural heterogeneities. Nano Lett..

[86] Wu S., Sun S., Ye J., Wang L., Zhang Y. (2025). Capillary-driven 3D open fluidic networks for versatile continuous flow manipulation. Adv. Mater..

[87] Mukherjee S., Elsayed M.Y., Tawfik H.H., El-Gamal M.N. (2024). A fabrication method for realizing vertically aligned silicon nanowires featuring precise dimension control. Sensors.

[88] Wang S., Yang J., Deng G., Zhou S. (2024). Femtosecond laser direct writing of flexible electronic devices: A mini review. Materials.

[89] Li Z., Luo Y., Xie X., Yang J., Zhuang H., Huang Y. (2025). High-resolution copper micropatterning on flexible substrates via laser-assisted surface activation. J. Mater. Process. Technol..

[90] Sedghamiz E., Liu M., Wenzel W. (2022). Challenges and limits of mechanical stability in 3D direct laser writing. Nat. Commun..

[91] Jiao R., Wang R., Wang Y., Cheung Y.K., Chen X., Wang X., Deng Y., Yu H. (2023). Vertical serpentine interconnect-enabled stretchable and curved electronics. Microsyst. Nanoeng..

[92] Jian W., Chen Y., Feng X. (2025). 3D conformal curvy electronics: Design, fabrication, and application. ACS Nano.

[93] Zhao P., Lim Y.D., Li H.Y., Luca G., Tan C.S. (2021). Advanced 3D integration technologies in various quantum computing devices. IEEE Open J. Nanotechnol..

[94] Rosenberg D., Kim D., Das R., Yost D., Gustavsson S., Hover D., Krantz P., Melville A., Racz L., Samach G.O. (2017). 3D integrated superconducting qubits. npj Quantum Inf..

[95] Yamagishi K., Lee S., Yokota T., Someya T. (2026). Soft, flexible, and stretchable platforms for tissue-interfaced bioelectronics. Adv. Sci..

[96] Kim S., Kim K.S., Koo J. (2025). Soft, conformal tissue-electrode interfaces for bioelectronic devices: Material, fabrication strategies, and applications. Biomed. Eng. Lett..

[97] Sitti M., Ceylan H., Hu W., Giltinan J., Turan M., Yim S., Diller E. (2015). Biomedical applications of untethered mobile milli/microrobots. Proc. IEEE.

